# Hyperparameter Optimization for Tomato Leaf Disease Recognition Based on YOLOv11m

**DOI:** 10.3390/plants14050653

**Published:** 2025-02-21

**Authors:** Yong-Suk Lee, Maheshkumar Prakash Patil, Jeong Gyu Kim, Yong Bae Seo, Dong-Hyun Ahn, Gun-Do Kim

**Affiliations:** 1 Department of Food Science and Technology/Institute of Food Science, Pukyong National University, Busan 48513, Republic of Korea; dragston@pknu.ac.kr; 2 Industry University Cooperation Foundation, Pukyong National University, Busan 48513, Republic of Korea; mahesh@pukyong.ac.kr; 3 Department of Microbiology, Pukyong National University, Busan 48513, Republic of Korea; icrus0306@pukyong.ac.kr (J.G.K.); haehoo76@pknu.ac.kr (Y.B.S.)

**Keywords:** hyperparameter optimization, tomato leaf disease, YOLOv11, one-factor-at-a-time, random search

## Abstract

The automated recognition of disease in tomato leaves can greatly enhance yield and allow farmers to manage challenges more efficiently. This study investigates the performance of YOLOv11 for tomato leaf disease recognition. All accessible versions of YOLOv11 were first fine-tuned on an improved tomato leaf disease dataset consisting of a healthy class and 10 disease classes. YOLOv11m was selected for further hyperparameter optimization based on its evaluation metrics. It achieved a fitness score of 0.98885, with a precision of 0.99104, a recall of 0.98597, and a mAP@.5 of 0.99197. This model underwent rigorous hyperparameter optimization using the one-factor-at-a-time (OFAT) algorithm, with a focus on essential parameters such as batch size, learning rate, optimizer, weight decay, momentum, dropout, and epochs. Subsequently, random search (RS) with 100 configurations was performed based on the results of OFAT. Among them, the C47 model demonstrated a fitness score of 0.99268 (a 0.39% improvement), with a precision of 0.99190 (0.09%), a recall of 0.99348 (0.76%), and a mAP@.5 of 0.99262 (0.07%). The results suggest that the final model works efficiently and is capable of accurately detecting and identifying tomato leaf diseases, making it suitable for practical farming applications.

## 1. Introduction

Tomato (*Solanum lycopersicum*) is one of the most consumed vegetables and holds significant economic importance worldwide [1]. The genus *Solanum* originated from South America, and plants of this genus have been widely cultivated and diversified [2]. Tomato is characterized by compound leaves with serrated leaflets, covered in glandular trichomes [3]. Tomato is abundant in essential nutrients, notably vitamin C, potassium, folate, and vitamin K [4]. It serves as a significant dietary source of lycopene, a potential antioxidant associated with several health benefits, including reduced risk of heart disease and cancer [3]. Tomatoes exhibit high genetic diversity, with thirteen closely related species and four associated *Solanum* species constituting a separate group with different mating systems [4]. The genetic diversity of tomatoes has made them a model organism for research into fruit ripening, hormone activity, and vitamin production [4].

Tomato is extremely vulnerable to several pathogenic and pest-related challenges that considerably endanger global production. This includes bacterial diseases, such as bacterial spot; fungal diseases, including late blight, early blight, target spot, septoria leaf spot, leaf mold, and powdery mildew; viral diseases, like tomato yellow leaf curl virus and tomato mosaic virus; and pest infestations by organisms like spider mites [5,6]. These diseases can lower yield by up to 40%, depending on the severity and management strategies employed [7]. Their economic impact is significant, with annual losses estimated at billions of dollars worldwide [8]. Furthermore, the frequency of these diseases often necessitates heavy pesticide use, causing increasing production costs and environmental issues [9,10]. Developing effective diagnostic tools and disease-resistant strategies is essential for mitigating these issues and ensuring sustained production [11]. The accurate and early diagnosis of tomato diseases is critical to reducing yield losses and guaranteeing food security amid a rapidly increasing world population [12,13]. Traditional diagnostic methods, including professional visual inspection and laboratory-based pathogen identification, are often employed [14,15]. Nonetheless, these strategies are labor-intensive, time-consuming, and frequently require experienced workers, hence constraining their scalability and real-time application in extensive agricultural operations [1,16]. In recent years, deep learning techniques have emerged as powerful instruments for disease diagnosis, utilizing convolutional neural networks (CNNs) to analyze intricate image data with exceptional accuracy and speed [1,12,17]. These methods have significant advantages, such as automation, flexibility to various settings, and the ability to analyze large datasets, thereby delivering real-time insights with minimal human intervention [18]. This paradigm shift in disease diagnosis has the potential to transform agricultural practices by facilitating more efficient and sustainable crop management [11].

Deep learning methods for object detection are categorized into two-stage and one-stage methodologies [19]. Two-stage methods, such as Faster R-CNN, exhibit high accuracy but are computationally demanding, restricting their application in real-time scenarios [20]. One-stage methods, such as YOLO, directly predict class probabilities and bounding boxes in a single iteration, attaining real-time performance with acceptable accuracy [21]. The efficient architecture and quick processing capabilities of YOLO models render them suitable for various applications where swift analysis is essential [13]. For example, Liu and Wang propose an improved YOLOv3 model to detect tomato diseases and pests [22]. The improved YOLOv3 model achieved a 92.39% detection accuracy with a detection time of 20.39 ms, outperforming Faster R-CNN, SSD, and the original YOLOv4 model in both accuracy and speed. Samal et al. present the SBMYv3 model, an improved YOLOv3 architecture for detecting obscene images and identifying explicit regions [23]. The model uses a custom-generated GLOI dataset, enriched through Pix-2-Pix GAN-based augmentation. The proposed SBMYv3 achieves a 99.26% testing accuracy, 99.13% precision, and 99.13% IoU, outperforming the baseline while maintaining computational efficiency. Wang et al. introduce the scaled-YOLOv4, an enhanced version of YOLOv4 for real-time object detection, capable of scaling across different hardware [24]. The model is evaluated on the MS COCO dataset, achieving 55.5% AP (73.4% AP50) at 16 FPS with YOLOv4-large and 22.0% AP (42.0% AP50) at 443 FPS with YOLOv4-tiny. Chen et al. introduce a YOLOv4-based system for detecting scale pests in agricultural environments [25]. YOLOv4 is compared to Faster R-CNN and SSD, outperforming them with 100% accuracy for mealybugs, 89% for Coccidae, and 97% for Diaspididae. Kim et al. introduce ECAP-YOLO, an enhanced YOLOv5 model for small object detection in aerial images [26]. The proposed ECAP-YOLO achieves significant improvements: a 6.9% mAP increase on VEDAI, 5.4% for small cars on xView, 2.7% for small vehicles and ships on DOTA, and 2.4% for small cars on Arirang. It outperforms the original YOLOv5 in accuracy and efficiency. Chen et al. introduce an improved YOLOv5s-based detection model (YOLO-COF) for Camellia oleifera fruit detection in occluded environments [27]. The proposed YOLO-COF model achieves a mAP of 94.10% with a frame rate of 74.8 FPS and a compact model size of 27.1 MB. Wang et al. introduce an improved YOLOv6 model for detecting tomato leaf diseases in natural environments [28]. The improved YOLOv6 achieves significant performance enhancements, with a precision of 92.9%, recall of 95.2%, F1-score of 94.0%, and mAP of 93.8% on a tomato leaf disease dataset, outperforming YOLOX, YOLOv5, YOLOv6, YOLOv7, and YOLOv8 models. Norkobil Saydirasulovich et al. propose an improved YOLOv6-based fire detection system for smart city environments [29]. The improved YOLOv6 demonstrates robust performance, achieving a precision of 93.48%, a recall of 28.29%, and a mAP of 39.50% for small fire target detection, outperforming Faster R-CNN and YOLOv3. Yang et al. introduce Maize-YOLO, an improved YOLOv7-based model for real-time detection of maize pests [30]. The model achieved a mAP of 76.3%, a recall of 77.3%, and an FPS of 67, outperforming other state-of-the-art detection models while maintaining computational efficiency. Liu et al. propose an improved YOLOv7-KCC model for tree species classification in shelterbelts using UAV-captured RGB images [31]. The YOLOv7-KCC model achieves a mAP@0.5 of 98.91%, significantly outperforming Faster R-CNN, SSD, YOLOv4, and the original YOLOv7 by 5.71%, 5.97%, 7.86%, and 3.69%, respectively. Wang et al. propose ALF-YOLO, an enhanced version of YOLOv8 for ship detection in complex maritime environments [32]. ALF-YOLO achieved significant performance improvements, with the mAP@0.5 increasing to 99.1% on the Seaships datasets, representing a 0.41% gain, and to 92.7% on the McShips dataset, reflecting 0.43% improvements. Fang et al. introduce the CCS-YOLOv8, an enhanced version of YOLOv8 for livestock detection in complex grassland environments [33]. The CCS-YOLOv8 achieved significant performance improvements, with the mAP@0.5 increasing to 84.4% on the Qinghai livestock dataset, representing a 5.8% gain, and the mAP@0.75 improving to 60.3%, reflecting a 6.6% improvement.

Hyperparameter optimization in deep learning refers to the process of selecting the optimal set of hyperparameters, which are configuration settings external to the model, such as learning rate, batch size, number of layers, and activation functions [34,35]. Unlike model parameters, which are learned during training, hyperparameters must be predefined and significantly impact the model’s performance [36]. Proper optimization is essential to ensure the model achieves high accuracy, generalization, and efficient convergence [37]. The necessity of hyperparameter optimization arises from the fact that poorly chosen hyperparameters can lead to suboptimal training, overfitting, or underfitting [38]. The hyperparameters often interact in complex ways, making manual tuning impractical, especially in deep networks with numerous configurations [39]. Advanced optimization techniques, such as grid search, random search, and Bayesian optimization, automate this process, providing systematic approaches to identify the best hyperparameters for a specific dataset and task [40]. In recent studies, hyperparameter optimization has been extensively applied to improve the performance of deep learning models in agriculture. For example, Ramos et al. optimized the YOLOv8 model for smoke and wildfire detection to enhance agricultural safety [41]. By utilizing a one-factor-at-a-time (OFAT) approach followed by random search (RS), key hyperparameters such as learning rate, batch size, and weight decay were fine-tuned. The optimized model demonstrated a 1.39% increase in precision, a 1.48% increase in recall, and a 5.09% improvement in mAP@0.5:0.95, showcasing its robustness in detecting smoke and fire in real-world agricultural settings. Solimani et al. addressed challenges in tomato plant phenotyping by optimizing YOLOv8 for the detection of phenotypic traits, including flowers, fruits, and nodes [42]. The improved YOLOv8 achieved superior performance in detecting small objects, demonstrating significant accuracy gains for real-time tomato trait recognition.

In our previous study [43], an improved YOLOv5m model was developed for tomato leaf disease recognition using advanced soft attention modules and a Bi-directional Feature Pyramid Network (BiFPN). The study integrated C3NN modules with various attention mechanisms, including convolutional block attention module (CBAM), squeeze and excitation network (SE), efficient channel attention (ECA), and coordinate attention (CA), into the backbone and neck of YOLOv5m. This modification significantly enhanced feature representation and multi-scale feature fusion, resulting in improved recognition accuracy. The optimized model achieved a precision of 87.76%, recall of 87.20%, F1-score of 87.48, mAP@0.5 of 90.40%, and mAP@0.5:0.95 of 68.80%, demonstrating its effectiveness for detecting tomato leaf diseases in diverse environments.

To further improve the recognition performance of tomato leaf diseases, this study introduces three key enhancements:

1. Dataset Expansion: The dataset size was increased approximately fourfold to include a broader range of disease samples, improving the model’s robustness and generalization capabilities.

2. Hyperparameter Optimization: An extensive hyperparameter optimization process was conducted, including fine-tuning parameters such as learning rate, batch size, and weight decay. This optimization ensured efficient convergence and minimized overfitting or underfitting.

3. YOLOv11m Implementation: The newly proposed YOLOv11m model was employed, leveraging advanced architectural improvements to enhance detection precision and computational efficiency.

The experimental results demonstrate that the enhanced YOLOv11m model outperforms previous versions, achieving superior accuracy and robustness in recognizing tomato leaf diseases under real-world conditions.

## 2. Materials and Methods

### 2.1. Improved Tomato Leaf Disease Dataset

**Figure 1:**
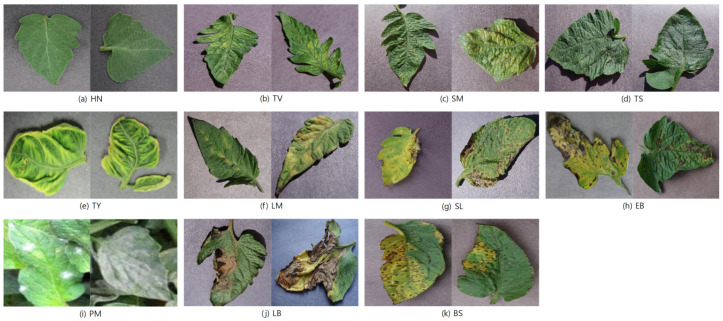
Sample images of healthy and infected tomato leaves. (Abbreviations: HN—healthy; TV—tomato mosaic virus; SM—spider mites two-spotted spider mite; TS—target spot; TY—tomato yellow leaf furl virus; LM—leaf mold; SL—septoria leaf spot; EB—early blight; PM—powdery mildew; LB—late blight; BS—bacteria spot).

In our previous study [43], the tomato leaf disease dataset was created from the tomato disease multiple sources in the Kaggle open repository (http://www.kaggle.com/datasets/cookiefinder/tomato-disease-multiple-sources, accessed on 30 December 2023) [44]. The tomato disease multiple sources comprised images from PlantVillage and Taiwan tomato leaves, which were augmented using rotations at multiple angles, mirroring, noise injection, flipping, etc. The tomato leaf disease dataset comprised 5500 images covering 10 diseases and 1 healthy class. To create the improved tomato leaf disease dataset, 22,000 images (2000 per class) were randomly selected from the tomato disease multiple sources. Samples of each class are shown in Figure 1. During the image collection process, poor-quality images were excluded. The selected images were augmented with rotations at various angles, as well as brightness adjustment and contrast adjustment. Each tomato leaf disease image was manually annotated using the labeling tool LabelImg (v1.8.1, Label Studio). Finally, a total of 22,000 images were randomly shuffled and divided into training, validation, and test sets in an 8:1:1 ratio, ensuring that neither the validation nor the test sets underwent augmentation. Detailed information about the dataset is shown in Table 1.

### 2.2. YOLOv11 Model

YOLOv11, unveiled at the YOLO Vision 2024 conference, represents a significant advancement in real-time object detection technology [45]. This latest iteration of the YOLO series builds upon its predecessors, YOLOv9 and YOLOv10, introduced in early 2024 [46]. YOLOv11 incorporates significant upgrades in both architecture and training methodologies, pushing the boundaries of speed, efficiency, and accuracy. The model is versatile across various computer vision tasks, including object recognition, image classification, pose estimation, instance segmentation, and oriented bounding box (OBB) detection [45]. YOLOv11′s refined architecture allows it to capture more nuanced details while maintaining a lean parameter count, thereby improving accuracy across diverse applications [15].

A significant improvement in the YOLOv11 is the introduction of the C2PSA (Convolutional block with Parallel Spatial Attention), SPPF (Spatial Pyramid Pooling—Fast), and C3k2 (Cross-Stage Partial Block with kernel size 2) blocks. As a result, YOLOv11 enhances the model’s ability to focus on critical regions while ignoring irrelevant areas. These blocks significantly boost efficiency by reducing computational complexity through optimized kernel operations, making the model well-suited for real-time applications. Additionally, the integration of transformer-like self-attention layers allows YOLOv11 to effectively capture long-range dependencies, further enhancing its accuracy and robustness.

**Figure 2:**
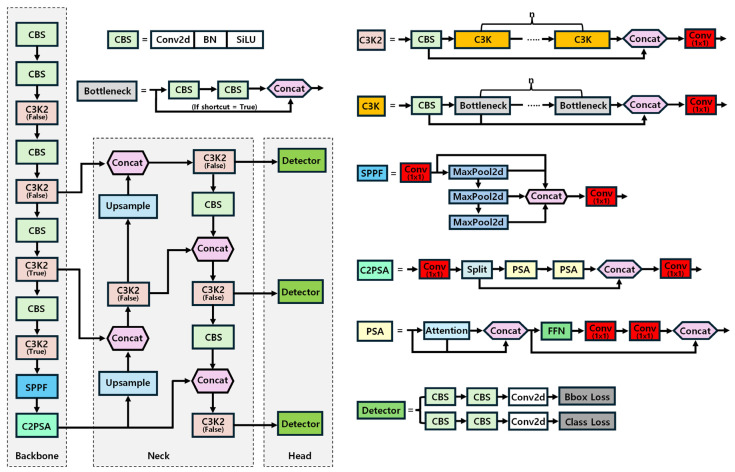
Model architecture of YOLOv11.

The architecture of YOLOv11 comprises three key components: the head, neck, and backbone. The backbone is responsible for extracting features from the input image at various scales. It typically consists of convolutional layers that progressively decrease spatial dimensions while enhancing the depth of feature maps. Advanced versions may incorporate residual connections and attention mechanisms to further enhance feature representation. The neck bridges the backbone and the head, aggregating multi-scale features for better object detection across varying object sizes. It often includes structures like Feature Pyramid Networks (PANs) to improve multi-scale feature fusion and ensure robust object detection across different sizes. The head is tasked with generating the final predictions, including object localization and bounding box regression [45]. It usually comprises several convolutional layers that process the aggregated features from the neck, outputting the probability scores and coordinates for detected objects. The design may vary to optimize for speed and accuracy. The structure of YOLOv11 is shown in Figure 2. It is available in various variants, such as YOLOv11n, YOLOv11s, YOLOv11m, YOLOv11l, and YOLOv11x. These variants support core functionalities like training, validation, inference, and export.

### 2.3. Evaluation Metrics

To verify the effectiveness of the model, this study uses the following evaluation metrics to measure detection performance: precision, recall, and F1-score, mean average precision (mAP).

Precision refers to the model’s ability to recognize only the relevant objects. A True Positive (TP) occurs when the model correctly predicts the diseased class, and a False Positive (FP) occurs when the model incorrectly predicts an object as diseased class. Precision is calculated as shown in Equation (1):

(1) $$Precision=\frac{TP}{TP+FP}$$

Recall refers to the model’s ability to recognize all the relevant objects. A False Negative (FN) means the model fails to predict the diseased class when it is actually present. Recall is calculated as shown in Equation (2):

(2) $$Recall=\frac{TP}{TP+FN}$$

F1-score is a metric used to evaluate the performance of a classification model by integrating both precision and recall. It is particularly advantageous in scenarios where both FP and FN carry significant importance. The F1-score is calculated as shown in Equation (3):

(3) $$F1-score=2\times \frac{Precision\times Recall}{Precision+Recall}$$

mAP is a comprehensive metric that assesses the model’s ability to detect and locate targets, taking into account performance differences among various categories. The mAP is calculated as shown in Equation (4):

(4) $$mAP=\frac{1}{n}\sum_{i=1}^{n}{AP}_{i},$$

where *n* represents the number of target categories and *AP_i_* the average precision for each category *i*. The average precision (AP) is obtained by calculating the area under the precision–recall (PR) curve.

Fitness is a comprehensive metric used to evaluate the overall performance of the model. It is typically defined as a weighted combination of various performance metrics, providing a single scalar value that represents the model’s effectiveness. The fitness is calculated as shown in Equation (5):

(5) Fitness=0.45×Precision+0.45×Recall+0.1×mAP@.5

This approach balances critical metrics, ensuring robust performance and mitigating overfitting by avoiding excessive optimization toward a single metric. The inclusion of weights (0.45 for precision and recall, 0.1 for mAP@.5) emphasizes application-specific priorities, while the scalar value simplifies hyperparameter optimization and facilitates result interpretation.

### 2.4. Workflow

#### 2.4.1. Fine-Tuning

Fine-tuning a YOLO model is a form of transfer learning, in which knowledge from a network pre-trained on a large-scale dataset (e.g., COCO) is adapted to a specific dataset [47]. Since training YOLO weights from scratch demands extensive computational resources and a massive number of labeled images, which are often unavailable in specific domains like agricultural imaging. Therefore, transfer learning offers an efficient solution to achieve high performance with limited data [48,49]. Various pretrained YOLOv11 variants were used for fine-tuning with the tomato leaf disease dataset. This fine-tuning process was performed under the following conditions: a batch size of 16, the SGD optimizer, a learning rate of 0.01, a weight decay of 0.0005, a momentum of 0.937, no dropout, and 100 epochs. The best model was selected through a comprehensive analysis of evaluation metrics, including fitness, loss function, and learning time. The selected model was subsequently employed for hyperparameter optimization.

#### 2.4.2. Hyperparameter Algorithms

The OFAT method involves adjusting a single factor while keeping all other factors constant [50]. This method systematically explores the effect of each factor on model performance. Using the OFAT method, one factor allows for the realization of the effect produced by each variable in isolation; however, interactions between factors may go undetected.

The RS method, on the other hand, involves selecting hyperparameter configurations randomly from a specified search space [40]. This method allows for efficient exploration of a large hyperparameter space without requiring exhaustive testing. In practice, RS often outperforms grid search, especially in high-dimensional spaces, and can discover optimal configurations with fewer iterations.

Comparing these methods, OFAT provides a structured approach that can be intuitive but may be less efficient in complex hyperparameter landscapes. RS, while less systematic, can more effectively explore the hyperparameter space, particularly when some parameters are more important than others. OFAT may struggle with parameter interactions, while RS can inadvertently capture these. In terms of computational efficiency, RS often requires fewer iterations to find good configurations. However, OFAT can provide clearer insights into the impact of individual parameters, which can be valuable for understanding model behavior.

#### 2.4.3. Hyperparameters Analyzed

Batch size in deep learning refers to the number of training examples utilized in one iteration during the model training process [49]. It is a critical hyperparameter that significantly affects the efficiency, speed, and performance of the training process [51]. The optimal batch size depends on factors such as dataset size, model complexity, and available computational resources [52].

Optimizer is an algorithm that adjusts the model’s parameters (weights and biases) to minimize the loss function during training. It guides the model towards its optimal performance by iteratively updating these parameters based on the computed gradients of the loss function. Optimizers play a crucial role in shaping the model’s learning process, balancing convergence speed and stability. They help overcome challenges such as slow convergence in areas with high curvature, reduce oscillations, and enable the algorithm to escape local minima more effectively. The choice of optimizer can significantly impact the model’s learning efficiency and final performance. The representative types of optimizers are as follows: Stochastic Gradient Descent (SGD), Adaptive Moment Estimation (Adam), Adamax, Nesterov-accelerated Adaptive Moment Estimation (NAdam), and Rectified Adaptive Moment Estimation (RAdam).

Learning rate determines the step size in each iteration of the optimization algorithm during model training [53]. It controls how much the model’s parameters are adjusted in response to the estimated error gradient [54]. An optimal learning rate ensures the model learns sufficiently from the training data to make meaningful parameter adjustments without overcorrecting or getting stuck in suboptimal solutions [55].

Weight decay, also known as L2 regularization, is a technique used to prevent overfitting by adding a penalty term to the loss function [56]. This regularization method helps improve generalization by reducing the model’s complexity and preventing it from fitting noise in the training data [57].

Momentum is an optimization technique used to accelerate convergence and improve the stability of gradient-based algorithms [58]. It incorporates a fraction of the previous update into the current update, effectively smoothing out parameter adjustments and allowing the optimizer to maintain velocity in consistent gradient directions [59]. This approach helps overcome issues like slow convergence in areas with high curvature, reduces oscillations, and enables the algorithm to escape local minima more effectively, ultimately leading to faster and more robust optimization [60].

Dropout is a regularization technique for neural networks that randomly deactivates a fraction of neurons and their connections during training [61]. This method prevents overfitting by reducing co-adaptation between neurons and effectively creating an ensemble of multiple sub-networks [62]. During inference, all neurons are retained, but their outputs are scaled to compensate for the dropout applied during training, resulting in improved generalization and robustness of the model [63].

Epochs refers to one complete pass through the entire training dataset through the learning algorithm [64]. During an epoch, the model sees every sample in the dataset, allowing it to adjust its weights and biases based on the calculated loss [65]. The number of epochs is a crucial hyperparameter that influences the model’s learning process, balancing between underfitting and overfitting [66]. Multiple epochs are typically used to optimize model performance, enabling the algorithm to refine its parameters iteratively and improve its ability to recognize patterns in the data [64].

In preparing this manuscript, ChatGPT (OpenAI, USA) was used to assist with proofreading and revisions.

## 3. Results and Discussions

### 3.1. Fine-Tuning Results Based on the Pretrained YOLOv11 Variants

To apply the pretrained YOLOv11 variants (YOLOv11n, YOLOv11s, YOLOv11m, YOLOv11l, and YOLOv11x) to the tomato leaf disease dataset, a fine-tuning was performed. The results of fine-tuning these YOLOv11 variants are shown in Table 2.

YOLOv11n achieved 6.3 FGLOPs and an image processing speed of 0.81 millisecond (ms), with a preprocess time of 0.11 ms, an inference time of 0.27 ms, and a postprocess time of 0.43 ms, a model size of 5.4 MB, and 1.519 h of training time during the training process. It achieved a fitness score of 0.98395, a precision of 0.98379, a recall of 0.98293, and a mAP@.5 of 0.98928 during the test.

YOLOv11s achieved 21.3 FGLOPs and an image processing speed of 0.80 ms, with a preprocess time of 0.03 ms, an inference time of 0.26 ms, and a postprocess time of 0.51 ms, a model size of 19.1 MB, and 1.539 h of training time during the training process. It achieved a fitness score of 0.98807, a precision of 0.98742, a recall of 0.98784, and a mAP@.5 of 0.99202 during the test.

YOLOv11m achieved 67.7 FGLOPs and an image processing speed of 0.95 ms, with a preprocess time of 0.05 ms, an inference time of 0.42 ms, and a postprocess time of 0.48 ms, a model size of 40.5 MB, and 1.861 h of training time during the training process. It achieved a fitness score of 0.98885, a precision of 0.99104, a recall of 0.98597, and a mAP@.5 of 0.99197 during the test.

YOLOv11l achieved 86.6 FGLOPs and an image processing speed of 0.84 ms, with a preprocess time of 0.0005 ms, an inference time of 0.45 ms, and a postprocess time of 0.39 ms, a model size of 51.2 MB, and 2.523 h of training time during the training process. It achieved a fitness score of 0.99008, a precision of 0.99259, a recall of 0.98682, and a mAP@.5 of 0.99350 during the test.

YOLOv11x achieved 194.5 FGLOPs and an image processing speed of 1.23 ms, with a preprocess time of 0.02 ms, an inference time of 0.73 ms, and a postprocess time of 0.48 ms, a model size of 114.4 MB, and 2.702 h of training time during the training process. It achieved a fitness score of 0.99162, a precision of 0.99177, a recall of 0.99112, and a mAP@.5 of 0.99321 during the test.

When the training and test processes for the various YOLOv11 variants were examined, most evaluation metrics tended to improve as the model size increased. The largest model, YOLOv11x, achieved the highest fitness score (0.99162) but required 2.702 h of training. The smallest model, YOLOv11n, achieved 0.98395 and required 1.519 h.

**Figure 3:**
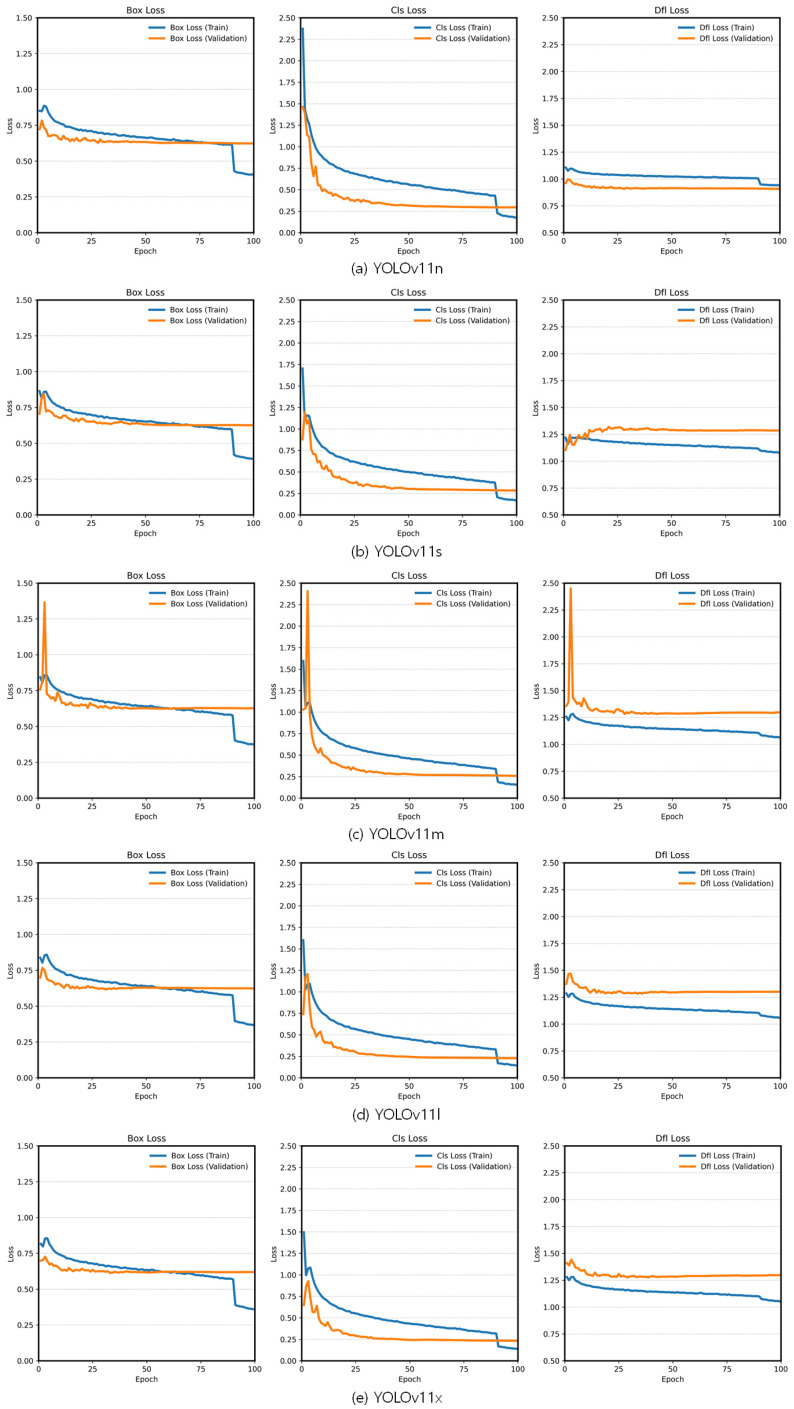
The loss functions of the YOLOv11 variants.

The medium model, YOLOv11m, achieved 0.98885 and required 1.861 h of training. The loss functions, such as box loss, classification (cls) loss, and dfl loss, for the YOLOv11 variants are shown in Figure 3. All loss functions rapidly or gradually decrease in the early stage of training, indicating efficient training. Moreover, comparing the train and validation curves shows no significant signs of overfitting, as the gap between these curves did not widen in the latter half of training. Among the YOLOv11 variants, YOLOv11m was determined to be the most suitable model for the hyperparameter optimization process due to its balance of learning time and high fitness value. Therefore, YOLOv11m was selected as the optimal candidate for further hyperparameter optimization, prioritizing a combination of performance and computational feasibility critical for real-world application in agricultural disease detection.

### 3.2. Hyperparameter Optimization

YOLOv11m was selected for the hyperparameter optimization during fine-tuning. Two types of algorithms, OFAT and RS, were used consecutively for hyperparameter optimization to improve the efficiency and accuracy of the tomato leaf disease recognition model. The most critical evaluation metric for model selection was the fitness score.

#### 3.2.1. OFAT

The OFAT method was performed as the first step in the hyperparameter optimization process to improve the performance of the tomato leaf disease recognition model. OFAT analyzed various values such as batch size, learning rate, weight decay, momentum, dropout, and epochs, while other hyperparameters remained unchanged during the analysis.

##### Batch Size

The detailed evaluation metrics for different batch sizes are presented in Table 3. A batch size of 16 (BS_16), which was used throughout the fine-tuning phase, served as the default for comparison.

With a batch size of 8 (BS_8), the model achieved an image processing speed of 1.03 ms, with a preprocess time of 0.03 ms, an inference time of 0.56 ms, and a postprocess time of 0.44 ms, and it required 3.108 h of training time during the training process. It achieved a fitness score of 0.98740, with a precision of 0.98645, a recall of 0.98730, and a mAP@.5 of 0.99208 (0.01% improvement) during the test.

With a batch size of 24 (BS_24), it achieved an image processing speed of 0.77 ms (18.95%), with a preprocess time of 0.07 ms, an inference time of 0.32 ms, and a postprocess time of 0.38 ms, and it required 1.305 h (29.88%) of training time during the training process. It achieved a fitness score of 0.99048 (0.16%), with a precision of 0.99233 (0.13%), a recall of 0.98824 (0.23%), and a mAP@.5 of 0.99224 (0.03%) during the test.

With a batch size of 32 (BS_32), it achieved an image processing speed of 0.74 ms (22.11%), with a preprocess time of 0.09 ms, an inference time of 0.35 ms, and a postprocess time of 0.30 ms, and it required 1.116 h (40.03%) of training time during the training process. It achieved a fitness score of 0.98878, with a precision of 0.98969, a recall of 0.98707 (0.11%), and a mAP@.5 of 0.99241 (0.04%) during the test.

With a batch size of 48 (BS_48), it achieved an image processing speed of 0.81 ms (14.74%), with a preprocess time of 0.06 ms, an inference time of 0.29 ms, and a postprocess time of 0.46 ms, and it required 1.003 h (46.10%) of training time during the training process. It achieved a fitness score of 0.98843, with a precision of 0.98911, a recall of 0.98681 (0.09%), and a mAP@.5 of 0.99272 (0.08%) during the test.

With a batch size of 64 (BS_64), it achieved an image processing speed of 0.81 ms (14.74%), with a preprocess time of 0.07 ms, an inference time of 0.29 ms, and a postprocess time of 0.45 ms, and it required 0.910 h (51.59%) of training time during the training process. It achieved a fitness score of 0.98922 (0.04%), with a precision of 0.99121 (0.02%), a recall of 0.98649 (0.05%), and a mAP@.5 of 0.99256 (0.06%) during the test.

With a batch size of 72 (BS_72), it achieved an image processing speed of 0.75 ms (21.05%), with a preprocess time of 0.003 ms, an inference time of 0.32 ms, and a postprocess time of 0.43 ms, and it required 0.862 h (53.68%) of training time during the training process. It achieved a fitness score of 0.98875, with a precision of 0.98901, a recall of 0.98793 (0.20%), and a mAP@.5 of 0.87309 during the test.

With a batch size of 80 (BS_80), it achieved an image processing speed of 0.82 ms (13.68%), with a preprocess time of 0.06 ms, an inference time of 0.31 ms, and a postprocess time of 0.45 ms, and it required 0.874 h (53.04%) of training time during the training process. It achieved a fitness score of 0.99144 (0.26%), with a precision of 0.99249 (0.15%), a recall of 0.99041 (0.45%), and a mAP@.5 of 0.99137 during the test.

With a batch size of 88 (BS_88), it achieved an image processing speed of 0.73 ms (23.16%), with a preprocess time of 0.01 ms, an inference time of 0.33 ms, and a postprocess time of 0.39 ms, and it required 0.823 h (55.78%) of training time during the training process. It achieved a fitness score of 0.98815, with a precision of 0.98767, a recall of 0.98780 (0.19%), and a mAP@.5 of 0.99188 during the test.

Among the tested configurations, only BS_24 and BS_80 achieved a fitness score exceeding 0.99000, with BS_80 recording the highest score of 0.99144. Compared to the default BS_16, BS_80 demonstrated approximately 0.26% improvement in fitness score. Additionally, BS_80 achieved notable gains in key metrics such as a 0.15% improvement in precision and a 0.45% improved improvement in recall. Among these metrics, recall was relatively greatly improved. The performance improvements associated with larger batch sizes might be attributed to enhanced gradient estimation stability, as larger batch sizes tend to produce more accurate gradient approximations, reducing variance during optimization [55]. However, performance did not improve linearly as batch size increased. It could be attributed to inadequate generalization, as overly large batch sizes can cause sharp minima in the loss landscape, negatively impacting model robustness [67]. Consequently, BS_80 was selected as the optimal batch size for hyperparameter optimization.

##### Optimizer

The detailed evaluation metrics for different optimizers are presented in Table 4. SGD, which was used throughout the batch size optimization phase, was established as the default for comparison analysis.

Adam achieved an image processing speed of 0.90 ms, with a preprocess time of 0.02 ms, an inference time of 0.38 ms, and a postprocess time of 0.50 ms, and required 0.932 h of training time during the training process. It achieved a fitness score of 0.93237, with a precision of 0.93105, a recall of 0.92667, and a mAP@.5 of 0.96391 during the test.

Adamax achieved an image processing speed of 0.89 ms, with a preprocess time of 0.02 ms, an inference time of 0.38 ms, and a postprocess time of 0.49 ms, and required 1.079 h of training time during the training process. It achieved a fitness score of 0.97563, with a precision of 0.97011, a recall of 0.97847, and a mAP@.5 of 0.98771 during the test.

AdamW achieved an image processing speed of 0.92 ms, with a preprocess time of 0.01 ms, an inference time of 0.36 ms, and a postprocess time of 0.55 ms, and required 0.866 h (0.92%) of training time during the training process. It achieved a fitness score of 0.89718, with a precision of 0.89851, a recall of 0.88714, and a mAP@.5 of 0.93736 during the test.

NAdam achieved an image processing speed of 0.97 ms, with a preprocess time of 0.00 ms, an inference time of 0.36 ms, and a postprocess time of 0.61 ms, and required 0.958 h of training time during the training process. It achieved a fitness score of 0.94336, with a precision of 0.94373, a recall of 0.93709, and a mAP@.5 of 0.96984 during the test.

RAdam achieved an image processing speed of 0.84 ms, with a preprocess time of 0.02 ms, an inference time of 0.38 ms, and a postprocess time of 0.44 ms, and required 0.952 h of training time during the training process. It achieved a fitness score of 0.93843, with a precision of 0.94815, a recall of 0.92201, and a mAP@.5 of 0.96862 during the test.

In conclusion, among the analyzed optimizers, Adamax achieved the highest fitness score of 0.97533. However, despite its superior metrics compared to other optimizers, Adamax did not outperform the default optimizer, SGD, in overall performance. SGD is widely recognized for its effectiveness in object detection models across various fields [68,69,70,71]. Therefore, SGD was confirmed as the optimal optimizer for hyperparameter optimization.

##### Learning Rate

The detailed evaluation metrics for different learning rates are presented in Table 5. The learning rate of 0.0100 (LR_0100), which was used throughout the optimizer optimization phase, was established as the default for comparison.

With a learning rate of 0.0001 (LR_0001), it achieved an image processing speed of 0.88 ms, with a preprocess time of 0.03 ms, an inference time of 0.33 ms, and a postprocess time of 0.52 ms, and it required 0.899 h of training time during the training process. It achieved a fitness score of 0.98347, with a precision of 0.98660, a recall of 0.97901, and a mAP@.5 of 0.98948 during the test.

With a learning rate of 0.0005 (LR_0005), it achieved an image processing speed of 0.82 ms, with a preprocess time of 0.02 ms, an inference time of 0.34 ms, and a postprocess time of 0.46 ms, and it required 0.895 h of training time during the training process. It achieved a fitness score of 0.98855, with a precision of 0.98964, a recall of 0.98675, and a mAP@.5 of 0.99175 during the test.

With a learning rate of 0.0010 (LR_0010), it achieved an image processing speed of 0.86 ms, with a preprocess time of 0.04 ms, an inference time of 0.32 ms, and a postprocess time of 0.50 ms, and it required 0.909 h of training time during the training process. It achieved a fitness score of 0.99211 (0.07% improvement), with a precision of 0.99085, a recall of 0.99312 (0.27%), and a mAP@.5 of 0.99319 (0.18%) during the test.

With a learning rate of 0.0050 (LR_0050), it achieved an image processing speed of 0.82 ms, with a preprocess time of 0.02 ms, an inference time of 0.34 ms, and a postprocess time of 0.46 ms, and it required 0.912 h of training time during the training process. It achieved a fitness score of 0.98991, with a precision of 0.99206, a recall of 0.98756, and a mAP@.5 of 0.99086 during the test.

With a learning rate of 0.0500 (LR_0500), it achieved an image processing speed of 0.81 ms (1.22%), with a preprocess time of 0.04 ms, an inference time of 0.31 ms, and a postprocess time of 0.46 ms, and it required 0.896 h of training time during the training process. It achieved a fitness score of 0.98423, with a precision of 0.98472, a recall of 0.98239, and a mAP@.5 of 0.99025 during the test.

In conclusion, among the evaluated learning rates, LR_0010 demonstrated the highest fitness score of 0.99211 (0.07% improvement), along with a 0.27% improved recall and a 0.18% improved mAP@.5. Among these improved metrics, recall showed a relatively greater improvement. All other learning rates showed lower values compared to the default LR_0100. Learning rates that are too small, such as LR_0001, often lead to slow convergence and may cause the model to get trapped in local minima due to insufficient parameter updates [72]. Conversely, excessively large learning rates, such as LR_0500, can result in unstable training dynamics, causing the model to overshoot ideal solutions and exhibit unpredictable performance, as evidenced by the decreased precision and recall. It is consistent with the concept that faster learning rates can cause sharp minima in the loss landscape, which negatively affects generalization performance [73]. Consequently, LR_0010 was selected as the optimal learning rate for hyperparameter optimization.

##### Weight Decay

The detailed evaluation metrics for different weight decays are presented in Table 6. The weight decay of 0.00050 (WD_00050), which was utilized throughout the learning rate optimization phase, was set as the default for comparison analysis.

With a weight decay of 0.00001 (WD_00001), it achieved an image processing speed of 0.84 ms (2.33%), with a preprocess time of 0.06 ms, an inference time of 0.32 ms, and a postprocess time of 0.46 ms, and it required 0.904 h (0.55%) of training time during the training process. It achieved a fitness score of 0.99062, with a precision of 0.98995, a recall of 0.99098, and a mAP@.5 of 0.99203 during the test.

With a weight decay of 0.00005 (WD_00005), it achieved an image processing speed of 0.88 ms, with a preprocess time of 0.01 ms, an inference time of 0.34 ms, and a postprocess time of 0.53 ms, and it required 0.901 h (0.88%) of training time during the training process. It achieved a fitness score of 0.98768, with a precision of 0.98582, a recall of 0.98860, and a mAP@.5 of 0.99203 during the test.

With a weight decay of 0.00010 (WD_00010), it achieved an image processing speed of 0.90 ms, with a preprocess time of 0.01 ms, an inference time of 0.36 ms, and a postprocess time of 0.53 ms, and it required 0.903 h (0.66%) of training time during the training process. It achieved a fitness score of 0.98953, with a precision of 0.99091 (0.01%), a recall of 0.98786, and a mAP@.5 of 0.99186 during the test.

With a weight decay of 0.00100 (WD_00100), it achieved an image processing speed of 0.73 ms (15.12%), with a preprocess time of 0.02 ms, an inference time of 0.35 ms, and a postprocess time of 0.36 ms, and it required 0.918 h of training time during the training process. It achieved a fitness score of 0.98990, with a precision of 0.99142 (0.06%), a recall of 0.98796, and a mAP@.5 of 0.99175 during the test.

With a weight decay of 0.00500 (WD_00500), it achieved an image processing speed of 0.87 ms, with a preprocess time of 0.01 ms, an inference time of 0.35 ms, and a postprocess time of 0.51 ms, and it required 0.918 h of training time during the training process. It achieved a fitness score of 0.99090, with a precision of 0.99238 (0.15%), a recall of 0.989280, and a mAP@.5 of 0.99159 during the test.

With a weight decay of 0.01000 (WD_01000), it achieved an image processing speed of 0.88 ms, with a preprocess time of 0.04 ms, an inference time of 0.33 ms, and a postprocess time of 0.51 ms, and it required 0.901 h (0.88%) of training time on the training process. It achieved a fitness score of 0.99152, with a precision of 0.98982, a recall of 0.99293, and a mAP@.5 of 0.99283 during the test.

With a weight decay of 0.05000 (WD_05000), it achieved an image processing speed of 0.91 ms, with a preprocess time of 0.01 ms, an inference time of 0.34 ms, and a postprocess time of 0.56 ms, and it required 0.918 h of training time during the training process. It achieved a fitness score of 0.96913, with a precision of 0.97066, a recall of 0.96466, and a mAP@.5 of 0.98237 during the test.

In conclusion, among the tested configurations, WD_00001, WD_00500, and WD_01000 achieved a fitness score exceeding 0.99000. However, none of these surpassed the default WD_00050 in overall fitness. For the SGD optimizer, weight decay serves as a regularization technique that suppresses large weights and influences key hyperparameters, such as precision and recall [74,75]. In this study, evaluating weight decay variations under SGD revealed that precision values tended to increase for certain models, such as WD_00010, WD_00100, and WD_00500, while recall values tended to decrease across all models. Consequently, default WD_00050 was selected as the optimal weight decay for hyperparameter optimization.

##### Momentum

The detailed evaluation metrics for different momentums are presented in Table 7. A momentum of 0.937 (MMT_937), which was used throughout the weight decay optimization phase, was set as the default for comparison analysis.

With a momentum of 0.859 (MMT_859), it achieved an image processing speed of 0.79 ms (8.14%), with a preprocess time of 0.01 ms, an inference time of 0.36 ms, and a postprocess time of 0.42 ms, and it required 0.906 h (0.33%) of training time during the training process. It achieved a fitness score of 0.99037, with a precision of 0.99132 (0.05%), a recall of 0.98887, and a mAP@.5 of 0.99285 during the test.

With a momentum of 0.885 (MMT_885), it achieved an image processing speed of 0.83 ms (3.49%), with a preprocess time of 0.02 ms, an inference time of 0.34 ms, and a postprocess time of 0.47 ms, and it required 0.904 h (0.55%) of training time during the training process. It achieved a fitness score of 0.98817, with a precision of 0.98983, a recall of 0.98585, and a mAP@.5 of 0.99118 during the test.

With a momentum of 0.911 (MMT_911), it achieved an image processing speed of 0.83 ms (3.49%), with a preprocess time of 0.01 ms, an inference time of 0.36 ms, and a postprocess time of 0.46 ms, and it required 0.903 h (0.66%) of training time during the training process. It achieved a fitness score of 0.98730, with a precision of 0.98749, a recall of 0.98606, and a mAP@.5 of 0.99199 during the test.

With a momentum of 0.963 (MMT_963), it achieved an image processing speed of 0.92 ms, with a preprocess time of 0.01 ms, an inference time of 0.36 ms, and a postprocess time of 0.55 ms, and it required 0.909 h of training time during the training process. It achieved a fitness score of 0.98697, with a precision of 0.98248, a recall of 0.99037, and a mAP@.5 of 0.99189 during the test.

With a momentum of 0.989 (MMT_989), it achieved an image processing speed of 0.87 ms, with a preprocess time of 0.01 ms, an inference time of 0.36 ms, and a postprocess time of 0.50 ms, and it required 0.906 h (0.33%) of training time during the training process. It achieved a fitness score of 0.99049, with a precision of 0.99127 (0.04%), a recall of 0.98963, and a mAP@.5 of 0.99081 during the test.

In conclusion, among the tested configurations, MMT_989 and MMT_859 achieved a fitness score exceeding 0.99000. However, compared to the default MMT_937, no model had improved fitness values. For the SGD optimizer, momentum controls the convergence speed of SGD by incorporating the gradient values of the previous step, and it is known to affect key hyperparameters such as precision, recall, and especially training time [76,77]. In this study, varying momentum values under SGD generally led to decreased training times across most models. Regarding precision, an increase was observed only at the two extreme values, such as MMT_989 and MMT_859. However, for recall, all models displayed decreased results. Consequently, the default MMT_937 was selected as the optimal momentum for hyperparameter optimization.

##### Dropout

The detailed evaluation metrics for different dropouts are presented in Table 8. The dropout of 0.0 (DO_0), which was used throughout the momentum optimization phase, was set as the default for comparison.

Under all tested dropout conditions, the results remained identical to those of the default, DO_0. Dropout is a well-known regularization technique used to prevent overfitting and is generally effective in complex or overfitting-prone situations [61]. These findings suggest that the current dataset is sufficiently large and balanced; thus, varying the dropout did not affect the results. Consequently, the default DO_0 was selected as the optimal dropout for hyperparameter optimization.

##### Epoch

The detailed evaluation metrics for different epochs are presented in Table 9. The epoch of 100 (EPO_100), which was used throughout the dropout optimization phase, was set as the default for comparison analysis.

With an epoch of 150 (EPO_150), it achieved an image processing speed of 0.84 ms (2.33%), with a preprocess time of 0.05 ms, an inference time of 0.32 ms, and a postprocess time of 0.47 ms, and it required 1.335 h of training time during the training process. No early stopping occurred. It achieved a fitness score of 0.99171, with a precision of 0.99246 (0.16% improvement), a recall of 0.98939, and a mAP@.5 of 0.99202 during the test.

With an epoch of 200 (EPO_200), it achieved an image processing speed of 0.83 ms (3.49%), with a preprocess time of 0.01 ms, an inference time of 0.36 ms, and a postprocess time of 0.46 ms, and it required 1.238 h of training time during the training process. Training stopped early at the 140th epoch. It achieved a fitness score of 0.98994, with a precision of 0.99099, a recall of 0.98827, and a mAP@.5 of 0.99273 during the test.

With an epoch of 250 (EPO_250), it achieved an image processing speed of 0.92 ms, with a preprocess time of 0.01 ms, an inference time of 0.36 ms, and a postprocess time of 0.55 ms, and it required 1.482 h of training time during the training process. Training stopped early in the 166th epoch. It achieved a fitness score of 0.99126, with a precision of 0.99028, a recall of 0.99214, and a mAP@.5 of 0.99170 during the test.

With an epoch of 300 (EPO_300), it achieved an image processing speed of 0.80 ms (6.98%), with a preprocess time of 0.01 ms, an inference time of 0.35 ms, and a postprocess time of 0.44 ms, and it required 1.447 h of training time during the training process. Training stopped early in the 162nd epoch. It achieved a fitness score of 0.99119, with a precision of 0.99014, a recall of 0.99183, and a mAP@.5 of 0.99304 during the test.

With an epoch of 350 (EPO_350), it achieved an image processing speed of 0.81 ms (5.81%), with a preprocess time of 0.04 ms, an inference time of 0.32 ms, and a postprocess time of 0.45 ms, and it required 1.365 h of training time during the training process. Training stopped early at the 152nd epoch. It achieved a fitness score of 0.98995, with a precision of 0.98978, a recall of 0.98995, and a mAP@.5 of 0.99078 during the test.

With an epoch of 400 (EPO_400), it achieved an image processing speed of 0.86 ms, with a preprocess time of 0.00 ms, an inference time of 0.36 ms, and a postprocess time of 0.50 ms, and it required 2.093 h of training time during the training process. Training stopped early in the 231st epoch. It achieved a fitness score of 0.99171, with a precision of 0.99158, a recall of 0.99183, and a mAP@.5 of 0.99174 during the test.

In conclusion, among the tested configurations, EPO_150, EPO_250, EPO_300, and EPO_400 achieved a fitness score exceeding 0.99000. However, compared to the default EPO_100, no model demonstrated improved fitness values. These results suggest that increasing the number of epochs does not improve model performance because the model reaches saturation at around 100 epochs [64,66]. Consequently, the default EPO_100 was selected as the optimal epoch value for hyperparameter optimization.

#### 3.2.2. RS

The random search (RS) method was performed as the second step in the hyperparameter optimization process to improve the performance of the apple leaf disease recognition model. RS examined various hyperparameter configurations, such as weight decay, learning rate, batch size, and momentum, by randomly sampling values from predefined distributions. The learning rate and weight decay were set using a log-uniform distribution, while momentum was sampled using a uniform distribution. Table 10 provides a comprehensive overview of these hyperparameters, including their values and ranges. Other hyperparameters, such as the optimizer, dropout, and epochs, remained constant throughout each iteration. The 100 configurations (C1-C100) selected for RS are presented in Table 11, and their corresponding performance metrics are shown in Table 12 and Supplementary Data S1.

The C1 model was configured with a batch size of 24, a learning rate of 0.0025, a weight decay of 0.00004, and a momentum of 0.835. It achieved an image processing speed of 0.82 ms (4.65%), with a preprocess time of 0.03 ms, an inference time of 0.34 ms, and a postprocess time of 0.45 ms, and it required 1.335 h of training time during the training process. It achieved a fitness score of 0.99227 (0.02% improvement), with a precision of 0.99055, a recall of 0.99409 (0.10%), and a mAP@.5 of 0.99178 during the test.

The C47 model was configured with a batch size of 24, a learning rate of 0.0024, a weight decay of 0.00004, and a momentum of 0.840. It achieved an image processing speed of 0.93 ms, with a preprocess time of 0.05 ms, an inference time of 0.35 ms, and a postprocess time of 0.53 ms, and it required 1.421 h of training time during the training process. It achieved a fitness score of 0.99268 (0.06%), with a precision of 0.99190 (0.11%), a recall of 0.99348 (0.04%), and a mAP@.5 of 0.99262 during the test.

The C56 model was configured with a batch size of 24, a learning rate of 0.0027, a weight decay of 0.00002, and a momentum of 0.888. It achieved an image processing speed of 0.91 ms, with a preprocess time of 0.09 ms, an inference time of 0.36 ms, and a postprocess time of 0.46 ms, and it required 1.382 h of training time during the training process. It achieved a fitness score of 0.99224 (0.01%), with a precision of 0.99502 (0.42%), a recall of 0.98943, and a mAP@.5 of 0.99236 during the test.

The C95 model was configured with a batch size of 24, a learning rate of 0.0006, a weight decay of 0.00029, and a momentum of 0.977. It achieved an image processing speed of 0.94 ms, with a preprocess time of 0.01 ms, an inference time of 0.43 ms, and a postprocess time of 0.50 ms, and it required 1.439 h of training time during the training process. It achieved a fitness score of 0.99218 (0.01%), with a precision of 0.99243 (0.16%), a recall of 0.99198, and a mAP@.5 of 0.99200 during the test.

In conclusion, among the 100 different configurations, only four models, namely, the C1, C47, C56, and C95 models, showed higher fitness scores than the default OFAT.

The C1 model achieved an enhanced fitness score (0.99227, 0.02% improvement), primarily due to its highest recall value (0.99409, 0.10%) among the 100 configurations. The C47 model achieved the highest fitness score (0.99268, 0.06%) and F1-score (0.99269), driven by its second-highest recall (0.99348, 0.04%) and relatively balanced recall and precision (0.99190, 0.11%). The C56 model achieved an enhanced fitness score (0.99224, 0.01%), owing to its highest precision (0.99502, 0.42%). The C95 model also achieved an enhanced fitness score (0.99218, 0.01%), attributable to its relatively balanced precision (0.99243, 0.16%) and recall (0.99198).

**Figure 4:**
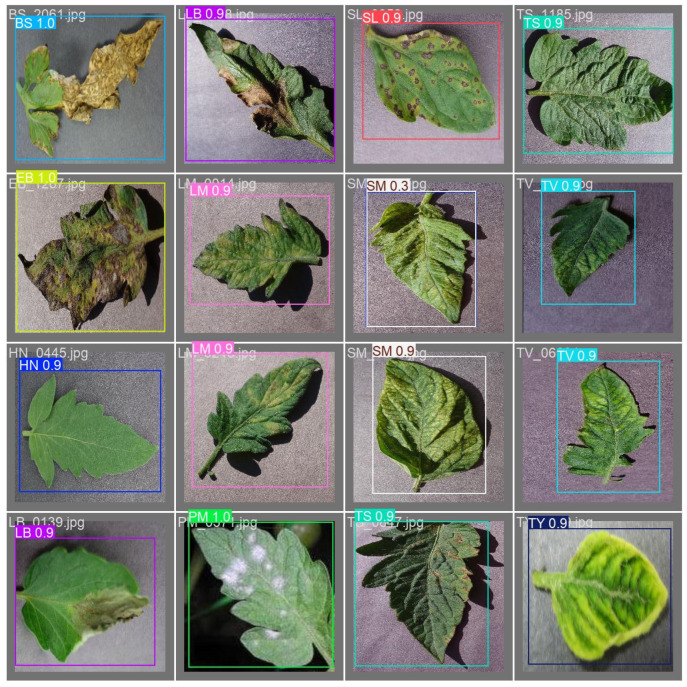
Detection results of the C47 model.

For the learning rate, the optimized configurations exhibited a relatively small range (0.0006 to 0.0027), promoting stable convergence. For the weight decay, the optimized configurations also fell within a small range (0.00002 to 0.00029), indicating effective training without overfitting. In contrast, momentum spanned a wider range in the optimized model. Regarding batch size, 24, which was the second highest value in OFAT analysis, appeared in all optimized configurations. The relatively modest batch size likely contributed to improved performance on the improved tomato leaf disease dataset used in this study. From these results, the C47 configuration was finally selected as the optimized model for tomato leaf disease recognition. Figure 4 illustrates detection results obtained using the C47 model.

### 3.3. Correlation Analysis Between Hyperparameters and Evaluation Metrics

**Figure 5:**
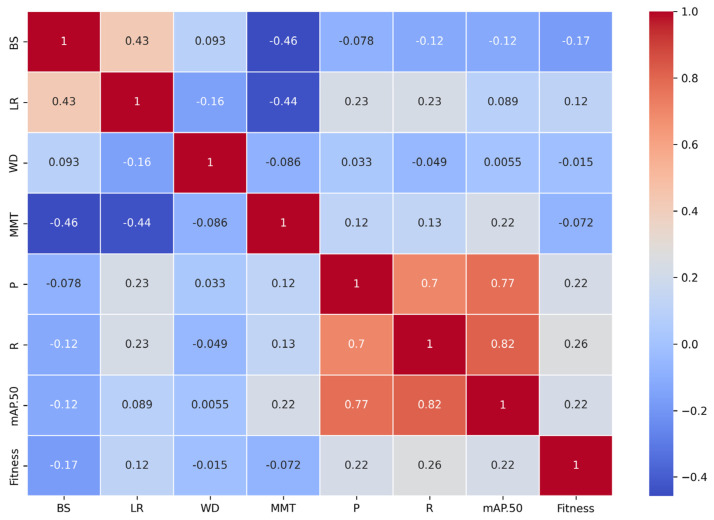
Correlation heatmap between hyperparameter and evaluation metrics.

The correlation analysis among hyperparameters such as weight decay, learning rate, batch size, and momentum and evaluation metrics such as mAP@.5, recall, precision, and fitness revealed distinct relationships that highlight the influence of these hyperparameters on model performance (Figure 5).

Batch size showed a moderate negative correlation with fitness (−0.17), precision (−0.078), and recall (−0.12), indicating that larger batch sizes tend to reduce gradient noise, which facilitates faster convergence but can lead to sharp minima in the loss landscape, negatively affecting generalization performance [55]. Learning rate exhibited weak positive correlations with precision (0.23), recall (0.23), and fitness (0.12), suggesting that smaller, stable learning rates facilitate marginal improvements in evaluation metrics while avoiding instability during training. This supports the hypothesis that learning rates affect the model’s capacity to escape local minima while maintaining stable parameter updates [78]. However, the correlation between learning rate and mAP@.5 was negligible (0.089), highlighting its limited direct effect on this metric. Weight decay had almost no significant correlation with precision (0.033), recall (−0.049), or fitness (−0.015), indicating that within the tested range, weight decay did not meaningfully impact model performance or generalization. This may imply that the model architecture already incorporates inherent regularization mechanisms (e.g., batch normalization and data augmentation), reducing the need for additional penalization on weight magnitudes. Momentum, on the other hand, displayed weak negative correlations with fitness (−0.072) and precision (0.12), along with a slight positive correlation with recall (0.13). This is consistent with findings that excessively high momentum can result in overshooting optimal solutions or underfitting certain data patterns [59].

Among the evaluation metrics, precision and recall showed a strong correlation (0.70), reflecting their complementary roles in detection tasks. These metrics also exhibited moderate positive correlations with fitness (0.22 for precision and 0.26 for recall), confirming their significant contributions to overall performance. mAP@.5, a key indicator of detection accuracy, demonstrated strong correlations with precision (0.77) and recall (0.82) while moderately influencing fitness (0.22). This finding underscores its importance in achieving balanced performance.

In conclusion, this analysis indicates that fitness, as a composite metric, is most strongly influenced by recall, followed by precision and mAP@.5. Hyperparameters such as batch size and momentum require careful tuning to optimize these metrics. These findings provide valuable guidance for refining hyperparameter configurations to achieve robust and accurate detection performance.

### 3.4. Comparison Experiments with Other Models

This research performed a comparative examination of the detection performance of the C47 model against other YOLO series, such as YOLOv3µ, YOLOv5m, YOLOv7, YOLOv8m, YOLOv9m, and YOLOv10m. The results are presented in Table 13.

YOLOv3µ (Y3µ) achieved an image processing speed of 1.16 ms, with a preprocess time of 0.02 ms, an inference time of 0.77 ms, and a postprocess time of 0.37 ms, and it required 2.220 h of training time during the training process. It achieved a fitness score of 0.98807, with a precision of 0.98564, a recall of 0.98971, and a mAP@.5 of 0.99157 during the test.

YOLOv5m (Y5m) achieved an image processing speed of 0.92 ms (1.08%), with a preprocess time of 0.04 ms, an inference time of 0.41 ms, and a postprocess time of 0.47 ms, and it required 1.601 h of training time during the training process. It achieved a fitness score of 0.98858, with a precision of 0.98785, a recall of 0.98885, and a mAP@.5 of 0.99069 during the test.

YOLOv7 (Y7) required 3.842 h of training time during the training process. It achieved a fitness score of 0.87554, with a precision of 0.90212, a recall of 0.83679, and a mAP@.5 of 0.93029 during the test.

YOLOv8m (Y8m) achieved an image processing speed of 0.87 ms (6.45%), with a preprocess time of 0.04 ms, an inference time of 0.45 ms, and a postprocess time of 0.38 ms, and it required 1.455 h of training time during the training process. It achieved a fitness score of 0.98821, with a precision of 0.98565, a recall of 0.98997, and a mAP@.5 of 0.99188 during the test.

YOLOv9m (Y9m) achieved an image processing speed of 0.93 ms, with a preprocess time of 0.04 ms, an inference time of 0.48 ms, and a postprocess time of 0.41 ms, and it required 2.199 h of training time during the training process. It achieved a fitness score of 0.98839, with a precision of 0.98612, a recall of 0.98994, and a mAP@.5 of 0.99160 during the test.

YOLOv10m (Y10m) achieved an image processing speed of 0.63 ms (32.26%), with a preprocess time of 0.07 ms, an inference time of 0.48 ms, and a postprocess time of 0.08 ms, and it required 2.322 h of training time during the training process. It achieved a fitness score of 0.98686, with a precision of 0.98575, a recall of 0.98678, and a mAP@.5 of 0.99218 during the test.

In conclusion, the comparative analysis demonstrated that the C47 model outperformed most YOLO series in terms of detection performance. It achieved a fitness score of 0.99268, surpassing models such as YOLOv3μ, YOLOv5m, YOLOv8m, and YOLOv9m, with slight but meaningful improvements in precision, recall, and mAP@.5. The C47 model showed particularly strong results in precision (0.99190) and recall (0.99348), underscoring its superior generalization capabilities. Furthermore, the C47 model maintained competitive training and inference speeds, making it both accurate and efficient. YOLOv11 not only maintains competitive speed and accuracy but also broadens the applicability of automated tomato leaf disease recognition in diverse agricultural environments [45]. These findings confirm that the C47 model provides a robust solution for tomato leaf disease recognition compared to other YOLO series models.

## 4. Conclusions and Future Works

In this study, to enhance performance of the tomato leaf disease recognition model using YOLOv11, the latest object detection model, we initially expanded the dataset used in our previous work (Table 1). The improved tomato leaf disease dataset consisted of 11 classes, 2000 images each, yielding a total of 22,000 images. Subsequently, we conducted hyperparameter optimization using one-factor-at-a-time (OFAT) and random search (RS). Among the hyperparameters analyzed by OFAT, such as batch size, optimizer, learning rate, weight decay, momentum, dropout, and epoch, improvements in performance were observed for batch size and learning rate compared to the default values. Based on these results, we performed RS with 100 configurations that varied batch size, learning rate, weight decay, and momentum. Of these, four models, C1, C47, C56, and C95, outperformed the OFAT default with the C47 model identified as the most optimal. Its hyperparameters included a batch size of 24, the SGD optimizer, a learning rate of 0.0024, a weight decay of 0.00004, a momentum of 0.840, a dropout of 0.0, and 100 epochs.

Looking to the future, we plan to conduct additional performance improvement research through architecture changes, such as integrating attention mechanisms and optimizing feature extraction layers, to enhance the efficiency and generalization of the C47 model. Additionally, we will conduct extensive ablation studies to systematically evaluate the impact of these modifications on detection accuracy, inference speed, and computational efficiency. Tomato leaf disease recognition improvement based on YOLOv11 will be carried out using all appropriate methodologies, including those used in our previous study. Moreover, to better capture real-world conditions, we will develop an augmented dataset enriched with images from operational tomato cultivation sites, thus ensuring a wider representation of disease appearances, environmental variables, and leaf morphologies. Concurrently, we plan to introduce specialized model variants, such as YOLOv11n, optimized for resource constrained environments, and YOLOv11x, designed for high-performance scenarios with abundant computational resource. We will benchmark the improved model against other state-of-the-art object detection frameworks to validate its superiority and robustness. Building on these advancements, we aim to develop a robust model suitable for deployment in urban farming applications and practical agricultural settings, facilitating real-world adoption and usability. To ensure successful real-world implementation, we will also explore hardware acceleration strategies and edge AI deployment for low-latency inference in resource-constrained environments.

## Figures and Tables

**Table 1:** Information on the used tomato leaf disease dataset.

Disease Name	Labels	Kaggle [44]		Previous Study [43]			This Study		
		Train	Valid	Train	Valid	Test	Train	Valid	Test
Healthy	HN	3051	806	400	50	50	1600	200	200
Tomato_mosaic_virus	TV	2153	584	400	50	50	1600	200	200
Spider_mites Two-spotted Spider_mite	SM	1747	435	400	50	50	1600	200	200
Target_Spot	TS	1827	457	400	50	50	1600	200	200
Tomato_Yellow_Leaf_Furl_Virus	TY	2039	498	400	50	50	1600	200	200
Leaf_Mold	LM	2754	739	400	50	50	1600	200	200
Septoria_leaf_spot	SL	2882	746	400	50	50	1600	200	200
Early_blight	EB	2455	643	400	50	50	1600	200	200
powdery Mildew	PM	1004	252	400	50	50	1600	200	200
Late_blight	LB	3113	792	400	50	50	1600	200	200
Bacteria_spot	BS	2826	732	400	50	50	1600	200	200
Total	-	25,851	6684	4400	550	550	17,600	2200	2200

**Table 2:** Performance of each YOLOv11 variant under fine-tuning with the tomato leaf disease.

Models	Process	Precision	Recall	F1-Score	mAP@.5	mAP@.5:.95	Fitness	Time (Hours)	Speed (ms)
YOLOv11n	Training	0.97792	0.97850	0.97821	0.98776	0.86416	0.97917	1.519	0.81
	Test	0.98379	0.98293	0.98336	0.98928	0.86988	0.98395	-	0.78
YOLOv11s	Training	0.98542	0.97826	0.98183	0.98886	0.86739	0.98255	1.539	0.80
	Test	0.98742	0.98784	0.98763	0.99202	0.87400	0.98807	-	0.95
YOLOv11m	Training	0.98505	**0.98263**	0.98384	0.99045	0.87092	0.98450	1.861	0.95
	Test	0.99104	0.98597	0.98850	0.99197	0.87666	0.98885	-	1.14
YOLOv11l	Training	**0.98690**	0.98128	**0.98408**	**0.99122**	**0.87247**	**0.98480**	2.523	0.84
	Test	**0.99259**	0.98682	0.98970	**0.99350**	0.87751	0.99008	-	1.31
YOLOv11x	Training	0.98482	0.98248	0.98365	0.98987	0.87206	0.98427	2.702	1.23
	Test	0.99177	**0.99112**	**0.99144**	0.99321	**0.87811**	**0.99162**	-	1.87

Bold indicates the best performance attained for each evaluation metric among all models. ‘-’, no evaluation.

**Table 3:** Performance metrics of different batch sizes.

Batch Size	Process	Precision	Recall	F1-Score	mAP@.5	mAP@.5:.95	Fitness	Time (Hours)	Speed (ms)
8	Training	0.98366	0.98250	0.98308	0.99130	0.87100	0.98390	3.108	1.03
	Test	0.98645	0.98730	0.98687	0.99208	**0.87695**	0.98740	-	1.24
16	Training	0.98505	0.98263	0.98384	0.99045	0.87092	0.98450	1.861	0.95
	Test	0.99104	0.98597	0.98850	0.99197	0.87666	0.98885	-	1.14
24	Training	0.98263	**0.98649**	0.98456	0.99108	0.86922	0.98521	1.305	0.77
	Test	0.99233	0.98824	0.99028	0.99224	0.87517	0.99048	-	1.00
32	Training	0.98528	0.98231	0.98379	0.99140	0.86879	0.98456	1.116	0.74
	Test	0.98969	0.98707	0.98838	0.99241	0.87551	0.98878	-	1.10
48	Training	0.98705	0.97781	0.98241	0.99120	**0.87156**	0.98331	1.003	0.81
	Test	0.98911	0.98681	0.98796	**0.99272**	0.87582	0.98843	-	1.10
64	Training	**0.98818**	0.98185	0.98500	**0.99165**	0.86990	0.98568	0.901	0.81
	Test	0.99121	0.98649	0.98884	0.99256	0.87461	0.98922	-	1.12
72	Training	0.98639	0.98601	0.98485	0.99091	0.87154	0.98545	0.862	0.75
	Test	0.98901	0.98793	0.98847	0.99130	0.87309	0.98875	-	1.23
80	Training	0.98735	0.98306	**0.98520**	0.99144	0.87064	**0.98583**	0.874	0.82
	Test	**0.99249**	**0.99041**	**0.99145**	0.99137	0.87309	**0.99144**	-	1.10
88	Training	0.98612	0.98173	0.98392	0.99152	0.86745	0.98469	0.823	0.73
	Test	0.98767	0.98780	0.98773	0.99188	0.87099	0.98815	-	1.01

Fixed hyperparameters: optimizer = SGD; learning rate = 0.01; weight decay = 0.00050; momentum = 0.937; no dropout; and epochs = 100. Bold indicates the best performance attained for each evaluation metric among all models. ‘-’, no evaluation.

**Table 4:** Performance metrics of different optimizers.

Optimizer	Process	Precision	Recall	F1-Score	mAP@.5	mAP@.5:.95	Fitness	Time (Hours)	Speed (ms)
SGD	Training	**0.98735**	**0.98306**	**0.98520**	**0.99144**	**0.87064**	**0.98583**	0.874	0.82
	Test	**0.99249**	**0.99041**	**0.99145**	**0.99137**	**0.87309**	**0.99144**	-	1.10
Adam	Training	0.94279	0.93403	0.93839	0.97474	0.83159	0.94204	0.932	0.90
	Test	0.93105	0.92667	0.92885	0.96391	0.82911	0.93237	-	1.19
Adamax	Training	0.97629	0.96717	0.97171	0.98791	0.85754	0.98335	1.079	0.89
	Test	0.97011	0.97847	0.97427	0.98771	0.86213	0.97563	-	1.05
AdamW	Training	0.92219	0.89974	0.91083	0.96372	0.80630	0.91624	0.866	0.92
	Test	0.89851	0.88714	0.89279	0.93736	0.79382	0.89718	-	1.03
NAdam	Training	0.94700	0.94129	0.94414	0.97978	0.84308	0.94771	0.958	0.97
	Test	0.94373	0.93709	0.94040	0.96984	0.83613	0.94336	-	1.06
RAdam	Training	0.94760	0.93541	0.94147	0.98027	0.83654	0.94538	0.952	0.84
	Test	0.94815	0.92201	0.93490	0.96862	0.83696	0.93843	-	1.04

Fixed hyperparameters: batch size = 80; learning rate = 0.0100; weight decay = 0.00050; momentum = 0.937; no dropout; and epochs = 100. Bold indicates the best performance attained for each evaluation metric among all models. ‘-’, no evaluation.

**Table 5:** Performance metrics of different learning rates.

Learning Rate	Process	Precision	Recall	F1-Score	mAP@.5	mAP@.5:.95	Fitness	Time (Hours)	Speed (ms)
0.0001	Training	0.98220	0.97163	0.97689	0.98854	0.84849	0.97808	0.899	0.88
	Test	0.98660	0.97901	0.98279	0.98948	0.86391	0.98347	-	0.97
0.0005	Training	0.98606	0.98215	0.98410	0.99126	0.86062	0.98482	0.895	0.82
	Test	0.98964	0.98675	0.98819	0.99175	0.86588	0.98855	-	1.07
0.0010	Training	0.98468	**0.98455**	0.98461	0.99034	0.86196	0.98519	0.909	0.86
	Test	0.99085	**0.99312**	**0.99198**	**0.99319**	0.86615	**0.99211**	-	1.07
0.0050	Training	0.98609	0.98233	0.98421	0.99070	0.86578	0.98486	0.912	0.82
	Test	0.99206	0.98756	0.98980	0.99086	0.86782	0.98991	-	1.10
0.0100	Training	**0.98735**	0.98306	**0.98520**	**0.99144**	**0.87064**	**0.98583**	0.874	0.82
	Test	**0.99249**	0.99041	0.99145	0.99137	**0.87309**	0.99144	-	1.10
0.0500	Training	0.98240	0.97295	0.97765	0.99063	0.86788	0.97897	0.896	0.81
	Test	0.98472	0.98239	0.98355	0.99025	0.87157	0.98423	-	1.05

Fixed hyperparameters: batch size = 80; optimizer = SGD; weight decay = 0.00050; momentum = 0.937; no dropout; and epochs = 100. Bold indicates the best performance attained for each evaluation metric among all models. ‘-’, no evaluation.

**Table 6:** Performance metrics of different weight decays.

Weight Decay	Process	Precision	Recall	F1-Score	mAP@.5	mAP@.5:.95	Fitness	Time (Hours)	Speed (ms)
0.00001	Training	**0.98883**	**0.98564**	**0.98723**	0.99074	0.86145	**0.98759**	0.904	0.84
	Test	0.98995	0.99098	0.99046	0.99203	0.86382	0.99062	-	1.12
0.00005	Training	0.98794	0.98453	0.98472	**0.99116**	0.86154	0.98538	0.901	0.88
	Test	0.98580	0.98860	0.98720	0.99199	0.86733	0.98768	-	1.02
0.00010	Training	0.98857	0.98351	0.98603	0.99103	0.86009	0.98654	0.903	0.90
	Test	0.99091	0.98796	0.98938	0.99186	0.86582	0.98953	-	1.03
0.00050	Training	0.98468	0.98455	0.98461	0.99034	0.86196	0.98519	0.909	0.86
	Test	0.99085	**0.99312**	**0.99198**	**0.99319**	0.86615	**0.99211**	-	1.07
0.00100	Training	0.98718	0.98171	0.98444	0.98976	0.85468	0.98498	0.918	0.73
	Test	0.99142	0.98796	0.98969	0.99175	0.86643	0.98990	-	1.03
0.00500	Training	0.98765	0.98227	0.98495	0.99100	**0.86265**	0.98556	0.918	0.87
	Test	**0.99238**	0.98928	0.99083	0.99159	0.87045	0.99090	-	1.09
0.01000	Training	0.98494	0.98469	0.98481	0.98930	0.86261	0.98526	0.901	0.88
	Test	0.98982	0.99293	0.99137	0.99283	**0.87170**	0.99152	-	1.12
0.05000	Training	0.97012	0.96706	0.96859	0.98925	0.84438	0.97066	0.918	0.91
	Test	0.97066	0.96466	0.96765	0.98237	0.86042	0.96913	-	1.04

Fixed hyperparameters: batch size = 80; optimizer = SGD; learning rate = 0.0010; momentum = 0.937; no dropout; and epochs = 100. Bold indicates the best performance attained for each evaluation metric among all models. ‘-’, no evaluation.

**Table 7:** Performance metrics of different momentums.

Momentum	Process	Precision	Recall	F1-Score	mAP@.5	mAP@.5:.95	Fitness	Time (Hours)	Speed (ms)
0.859	Training	0.98665	98044	0.98354	0.98988	0.85896	0.98418	0.906	0.79
	Test	**0.99132**	0.98887	0.99009	0.99285	0.86553	0.99037	-	1.22
0.885	Training	0.98479	0.98425	0.98452	0.99014	0.85877	0.98508	0.904	0.83
	Test	0.98983	0.98585	0.98784	0.99118	0.86357	0.98817	-	1.13
0.911	Training	0.98023	0.98380	0.98201	0.98986	0.85966	0.98280	0.903	0.83
	Test	0.98749	0.98606	0.98677	0.99199	0.86667	0.98730	-	1.12
0.937	Training	0.98468	**0.98455**	0.98461	0.99034	0.86196	0.98519	0.909	0.86
	Test	0.99085	**0.99312**	**0.99198**	**0.99319**	0.86615	**0.99211**	-	1.07
0.963	Training	**0.98732**	0.98293	**0.98512**	**0.99146**	0.86502	**0.98576**	0.909	0.92
	Test	0.98248	0.99037	0.98641	0.99189	0.86805	0.98697	-	1.13
0.989	Training	0.98598	0.98361	0.98479	0.99050	**0.86744**	0.98537	0.906	0.87
	Test	0.99127	0.98963	0.99045	0.99081	**0.87057**	0.99049	-	1.21

Fixed hyperparameters: batch size = 80; optimizer = SGD; learning rate = 0.0010; weight decay = 0.00050; no dropout; and epochs = 100. Bold indicates the best performance attained for each evaluation metric among all models. ‘-’, no evaluation.

**Table 8:** Performance metrics of different dropout.

Dropout	Process	Precision	Recall	F1-Score	mAP@.5	mAP@.5:.95	Fitness	Time (Hours)	Speed (ms)
0.0	Training	0.98468	0.98455	0.98461	0.99034	0.86196	0.98519	0.909	0.86
	Test	0.99085	0.99312	0.99198	0.99319	0.86615	0.99211	-	1.07
0.1	Training	0.98468	0.98455	0.98461	0.99034	0.86196	0.98519	0.909	0.86
	Test	0.99085	0.99312	0.99198	0.99319	0.86615	0.99211	-	1.07
0.3	Training	0.98468	0.98455	0.98461	0.99034	0.86196	0.98519	0.909	0.86
	Test	0.99085	0.99312	0.99198	0.99319	0.86615	0.99211	-	1.07
0.5	Training	0.98468	0.98455	0.98461	0.99034	0.86196	0.98519	0.909	0.86
	Test	0.99085	0.99312	0.99198	0.99319	0.86615	0.99211	-	1.07
0.7	Training	0.98468	0.98455	0.98461	0.99034	0.86196	0.98519	0.909	0.86
	Test	0.99085	0.99312	0.99198	0.99319	0.86615	0.99211	-	1.07

Fixed hyperparameters: batch size = 80; optimizer = SGD; learning rate = 0.0010; weight decay = 0.00050; momentum = 0.937; and epochs = 100. ‘-’, no evaluation.

**Table 9:** Performance metrics of different epochs.

Epoch	Process	Precision	Recall	F1-Score	mAP@.5	mAP@.5:.95	Fitness	Time (Hours)	Speed (ms)
100	Training	0.98468	**0.98455**	0.98461	0.99034	0.86196	0.98519	0.909	0.86
	Test	0.99085	**0.99312**	**0.99198**	**0.99319**	0.86615	**0.99211**	-	1.07
150	Training	**0.98885**	0.98327	**0.98605**	0.99092	0.86289	**0.98655**	1.335	0.84
	Test	**0.99246**	0.98939	0.99092	0.99202	0.86583	0.99103	-	1.11
200	Training	0.98757	0.98401	0.98579	0.99048	0.86649	0.98626	1.238	0.83
	Test	0.99099	0.98827	0.98963	0.99273	**0.87320**	0.98994	-	1.05
250	Training	0.98789	0.98308	0.98548	**0.99153**	**0.86662**	0.98597	1.482	0.92
	Test	0.99028	0.99214	0.99121	0.99170	0.86748	0.99126	-	1.02
300	Training	0.98730	0.98368	0.98549	0.99024	0.86624	0.98597	1.447	0.80
	Test	0.99014	0.99183	0.99098	0.99304	0.86990	0.99119	-	1.09
350	Training	0.98709	0.98154	0.98431	0.98967	0.86261	0.98485	1.365	0.81
	Test	0.98978	0.98995	0.98986	0.99078	0.86801	0.98995	-	1.04
400	Training	0.98801	0.98362	0.98581	0.99051	0.86185	0.98628	2.093	0.86
	Test	0.99158	0.99183	0.99170	0.99174	0.86475	0.99171	-	1.03

Fixed hyperparameters: batch size = 80; optimizer = SGD; learning rate = 0.0010; weight decay = 0.00050; momentum = 0.937; and no dropout. Bold indicates the best performance attained for each evaluation metric among all models. ‘-’, no evaluation.

**Table 10:** Overview of RS analysis hyperparameters and values.

Hyperparameter	Values/Ranges
Batch size	24, 80, 96, 128
Learning rate	0.0001~0.0100
Weight decay	0.00001~0.00100
Momentum	0.800~0.990

**Table 11:** Detailed RS analysis hyperparameter configurations.

Model	Batch Size	Learning Rate	Weight Decay	Momentum	Model	Batch Size	Learning Rate	Weight Decay	Momentum
C1	24	0.0025	0.00004	0.835	C51	80	0.0005	0.00075	0.823
C2	128	0.0088	0.00017	0.897	C52	80	0.0010	0.00010	0.861
C3	96	0.0004	0.00005	0.848	C53	128	0.0038	0.00003	0.806
C4	24	0.0020	0.00008	0.819	C54	128	0.0071	0.00008	0.833
C5	96	0.0012	0.00010	0.845	C55	96	0.0029	0.00002	0.898
C6	24	0.0005	0.00015	0.897	C56	24	0.0027	0.00002	0.888
C7	96	0.0001	0.00002	0.873	C57	80	0.0012	0.00014	0.871
C8	24	0.0015	0.00007	0.889	C58	80	0.0017	0.00061	0.88
C9	80	0.0089	0.00051	0.801	C59	128	0.0076	0.00007	0.809
C10	128	0.0019	0.00012	0.902	C60	128	0.0099	0.00007	0.826
C11	96	0.0033	0.00052	0.826	C61	24	0.0021	0.00002	0.919
C12	128	0.0024	0.00002	0.909	C62	24	0.0036	0.00004	0.938
C13	80	0.0078	0.00003	0.889	C63	80	0.0005	0.00009	0.856
C14	24	0.0002	0.00083	0.854	C64	96	0.0009	0.00005	0.881
C15	96	0.0006	0.00042	0.891	C65	128	0.0095	0.00012	0.816
C16	96	0.0047	0.00034	0.831	C66	128	0.0013	0.00020	0.955
C17	24	0.0004	0.00003	0.831	C67	24	0.0007	0.00001	0.944
C18	24	0.0008	0.00002	0.860	C68	24	0.0006	0.00030	0.969
C19	96	0.0004	0.00017	0.894	C69	24	0.0006	0.00001	0.988
C20	24	0.0016	0.00039	0.951	C70	24	0.0003	0.00001	0.937
C21	24	0.0008	0.00001	0.931	C71	80	0.0003	0.00003	0.960
C22	80	0.0003	0.00024	0.965	C72	96	0.0017	0.00002	0.842
C23	24	0.0047	0.00004	0.985	C73	80	0.0004	0.00073	0.865
C24	24	0.0002	0.00001	0.932	C74	80	0.0003	0.00043	0.826
C25	80	0.0002	0.00027	0.975	C75	128	0.0040	0.00003	0.854
C26	24	0.0051	0.00004	0.800	C76	128	0.0044	0.00008	0.832
C27	24	0.0001	0.00001	0.926	C77	96	0.0029	0.00004	0.905
C28	80	0.0002	0.00089	0.977	C78	96	0.0062	0.00003	0.896
C29	128	0.0060	0.00004	0.803	C79	96	0.0030	0.00002	0.890
C30	24	0.0029	0.00002	0.918	C80	24	0.0024	0.00014	0.884
C31	80	0.0010	0.00092	0.871	C81	80	0.0009	0.00037	0.847
C32	128	0.0075	0.00006	0.807	C82	80	0.0013	0.00099	0.853
C33	24	0.0034	0.00002	0.917	C83	128	0.0084	0.00006	0.812
C34	80	0.0010	0.00009	0.874	C84	128	0.0053	0.00007	0.823
C35	128	0.0098	0.00008	0.815	C85	24	0.0020	0.00005	0.927
C36	24	0.0014	0.00021	0.949	C86	128	0.0044	0.00009	0.910
C37	24	0.0007	0.00001	0.950	C87	24	0.0034	0.00004	0.913
C38	24	0.0006	0.00001	0.939	C88	24	0.0005	0.00009	0.867
C39	80	0.0003	0.00011	0.963	C89	96	0.0008	0.00005	0.857
C40	80	0.0021	0.00062	0.848	C90	96	0.0006	0.00012	0.879
C41	24	0.0040	0.00003	0.836	C91	96	0.0011	0.00016	0.837
C42	24	0.0064	0.00005	0.984	C92	128	0.0090	0.00022	0.804
C43	96	0.0001	0.00002	0.879	C93	24	0.0007	0.00024	0.955
C44	128	0.0002	0.00014	0.907	C94	128	0.0019	0.00018	0.943
C45	80	0.0014	0.00028	0.863	C95	24	0.0006	0.00029	0.977
C46	80	0.0002	0.00018	0.819	C96	24	0.0015	0.00006	0.970
C47	24	0.0024	0.00004	0.840	C97	24	0.0004	0.00001	0.980
C48	96	0.0055	0.00005	0.811	C98	24	0.0001	0.00001	0.989
C49	24	0.0001	0.00003	0.919	C99	80	0.0003	0.00050	0.960
C50	24	0.0017	0.00002	0.879	C100	96	0.0002	0.00003	0.840

**Table 12:** Best performance configurations of RS analyzed on training and test.

Model	Process	Precision	Recall	F1-Score	mAP@.5	mAP@.5:.95	Fitness	Time (Hours)	Speed (ms)
OFAT	Training	0.98468	0.98455	0.98461	0.99034	0.86196	0.98519	0.909	0.86
	Test	0.99085	0.99312	0.99198	**0.99319**	0.86615	0.99211	-	1.07
C1	Training	**0.99022**	0.98154	0.98586	0.99128	0.86481	0.98642	1.355	0.82
	Test	0.99055	**0.99409**	0.99232	0.99178	0.86894	0.99227	-	1.21
C47	Training	0.98609	0.98293	0.98451	0.99086	0.86359	0.98514	1.421	0.93
	Test	0.99190	0.99348	**0.99269**	0.99262	0.86842	**0.99268**	-	1.12
C56	Training	0.98831	**0.98650**	**0.98740**	**0.99150**	**0.86662**	**0.98782**	1.382	0.91
	Test	**0.99502**	0.98943	0.99222	0.99236	0.87094	0.99224	-	1.09
C95	Training	0.99021	0.98060	0.98538	0.99060	0.86548	0.98593	1.439	0.94
	Test	0.99243	0.99198	0.99220	0.99200	0.87179	0.99218	-	1.13

Fixed hyperparameters: optimizer = SGD; no dropout; epochs = 100. Bold indicates the best performance attained for each evaluation metric among all models. ‘-’, no evaluation.

**Table 13:** Performance of other models.

Model	Process	Precision	Recall	F1-Score	mAP@.5	mAP@.5:.95	Fitness	Time (Hours)	Speed (ms)
YOLOv3µ	Training	**0.99046**	0.98117	**0.98579**	0.99036	0.86732	**0.98627**	2.220	1.16
	Test	0.98564	0.98971	0.98767	0.99157	0.87301	0.98807	-	1.92
YOLOv5m	Training	0.98520	0.97657	0.98087	**0.99161**	**0.87153**	0.98196	1.601	0.92
	Test	0.98785	0.98885	0.98835	0.99069	0.87159	0.98858	-	0.97
YOLOv7	Training	0.91633	0.84273	0.87799	0.93863	0.77301	0.88544	3.842	-
	Test	0.90212	0.83679	0.86826	0.93029	0.75676	0.87554	-	-
YOLOv8m	Training	0.98597	0.97636	0.98114	0.98906	0.87060	0.98195	1.455	0.87
	Test	0.98565	0.98997	0.98781	0.99188	0.87443	0.98821	-	1.04
YOLOv9m	Training	0.98840	0.97812	0.98323	0.99115	0.86930	0.98405	2.199	0.93
	Test	0.98612	0.98994	0.98803	0.99160	**0.87643**	0.98839	-	1.16
YOLOv10m	Training	0.98359	0.97809	0.98083	0.99095	0.86954	0.98185	2.322	0.63
	Test	0.98575	0.98678	0.98626	0.99218	0.87218	0.98686	-	0.76
C47	Training	0.98609	**0.98293**	0.98451	0.99086	0.86359	0.98514	1.421	0.93
	Test	**0.99190**	**0.99348**	**0.99269**	**0.99262**	0.86842	**0.99268**	-	1.12

Bold indicates the best performance attained for each evaluation metric among all models. ‘-’, no evaluation.

## Data Availability

The data presented in this study are openly available in Tomato Disease Multiple Sources at https://www.kaggle.com/datasets/cookiefinder/tomato-disease-multiple-sources/data, accessed on 30 December 2023.

## Supplementary Materials

The following supporting information can be downloaded at https://www.mdpi.com/article/10.3390/plants14050653/s1, Data S1: Performance configurations of RS analyzed on training and test.

## References

[1] Jing J., Li S., Qiao C., Li K., Zhu X., Zhang L. (2023). A tomato disease identification method based on leaf image automatic labeling algorithm and improved YOLOv5 model. J. Sci. Food Agric..

[2] Panno S., Davino S., Caruso A.G., Bertacca S., Crnogorac A., Mandić A., Noris E., Matić S. (2021). A review of the most common and economically important diseases that undermine the cultivation of tomato crop in the mediterranean basin. Agronomy.

[3] Li N., Wu X., Zhuang W., Xia L., Chen Y., Wu C., Rao Z., Du L., Zhao R., Yi M. (2021). Tomato and lycopene and multiple health outcomes: Umbrella review. Food Chem..

[4] Sainju U.M., Dris R., Singh B. (2003). Mineral nutrition of tomato. Food Agric. Environ..

[5] Jones J.B., Zitter T.A., Momol T.M., Miller S.A. (2014). Compendium of Tomato Diseases and Pests.

[6] Kannan V.R., Bastas K.K., Devi R.S. (2015). 20 Scientific and Economic Impact of Plant Pathogenic Bacteria. Sustainable Approaches to Controlling Plant Pathogenic Bacteria.

[7] Jones J. (1991). Compendium of tomato diseases. American Phytopathological Society.

[8] Subedi B., Poudel A., Aryal S. (2023). The impact of climate change on insect pest biology and ecology: Implications for pest management strategies, crop production, and food security. J. Agric. Food Res..

[9] Van Der Werf H.M. (1996). Assessing the impact of pesticides on the environment. Agric. Ecosyst. Environ..

[10] Lykogianni M., Bempelou E., Karamaouna F., Aliferis K.A. (2021). Do pesticides promote or hinder sustainability in agriculture? The challenge of sustainable use of pesticides in modern agriculture. Sci. Total Environ..

[11] Shoaib M., Shah B., Ei-Sappagh S., Ali A., Ullah A., Alenezi F., Gechev T., Hussain T., Ali F. (2023). An advanced deep learning models-based plant disease detection: A review of recent research. Front. Plant Sci..

[12] Appe S.N., Arulselvi G., Balaji G. (2023). CAM-YOLO: Tomato detection and classification based on improved YOLOv5 using combining attention mechanism. PeerJ Comput. Sci..

[13] Qi J., Liu X., Liu K., Xu F., Guo H., Tian X., Li M., Bao Z., Li Y. (2022). An improved YOLOv5 model based on visual attention mechanism: Application to recognition of tomato virus disease. Comput. Electron. Agric..

[14] Zhang Y., Huang S., Zhou G., Hu Y., Li L. (2023). Identification of tomato leaf diseases based on multi-channel automatic orientation recurrent attention network. Comput. Electron. Agric..

[15] Alif M.A.R. (2024). YOLOv11 for Vehicle Detection: Advancements, Performance, and Applications in Intelligent Transportation Systems. arXiv.

[16] Buja I., Sabella E., Monteduro A.G., Chiriacò M.S., De Bellis L., Luvisi A., Maruccio G. (2021). Advances in plant disease detection and monitoring: From traditional assays to in-field diagnostics. Sensors.

[17] Touko Mbouembe P.L., Liu G., Park S., Kim J.H. (2024). Accurate and fast detection of tomatoes based on improved YOLOv5s in natural environments. Front. Plant Sci..

[18] Ferentinos K.P. (2018). Deep learning models for plant disease detection and diagnosis. Comput. Electron. Agric..

[19] Khan A.I., Quadri S., Banday S., Shah J.L. (2022). Deep diagnosis: A real-time apple leaf disease detection system based on deep learning. Comput. Electron. Agric..

[20] Ren S., He K., Girshick R., Sun J. Faster r-cnn: Towards real-time object detection with region proposal networks. Proceedings of the Annual Conference on Advances in Neural Information Processing Systems.

[21] Redmon J., Divvala S., Girshick R., Farhadi A. You only look once: Unified, real-time object detection. Proceedings of the IEEE Conference on Computer Vision and Pattern Recognition.

[22] Liu J., Wang X. (2020). Tomato diseases and pests detection based on improved Yolo V3 convolutional neural network. Front. Plant Sci..

[23] Samal S., Zhang Y.D., Gadekallu T.R., Nayak R., Balabantaray B.K. (2023). SBMYv3: Improved MobYOLOv3 a BAM attention-based approach for obscene image and video detection. Expert Syst..

[24] Wang C.-Y., Bochkovskiy A., Liao H.-Y.M. Scaled-YOLOv4: Scaling cross stage partial network. Proceedings of the IEEE/CVF Conference on Computer Vision and Pattern Recognition (CVPR).

[25] Chen J.-W., Lin W.-J., Cheng H.-J., Hung C.-L., Lin C.-Y., Chen S.-P. (2021). A smartphone-based application for scale pest detection using multiple-object detection methods. Electronics.

[26] Kim M., Jeong J., Kim S. (2021). ECAP-YOLO: Efficient channel attention pyramid YOLO for small object detection in aerial image. Remote Sens..

[27] Chen S., Zou X., Zhou X., Xiang Y., Wu M. (2023). Study on fusion clustering and improved YOLOv5 algorithm based on multiple occlusion of Camellia oleifera fruit. Comput. Electron. Agric..

[28] Wang Y., Zhang P., Tian S. (2024). Tomato leaf disease detection based on attention mechanism and multi-scale feature fusion. Front. Plant Sci..

[29] Norkobil Saydirasulovich S., Abdusalomov A., Jamil M.K., Nasimov R., Kozhamzharova D., Cho Y.-I. (2023). A YOLOv6-based improved fire detection approach for smart city environments. Sensors.

[30] Yang S., Xing Z., Wang H., Dong X., Gao X., Liu Z., Zhang X., Li S., Zhao Y. (2023). Maize-YOLO: A new high-precision and real-time method for maize pest detection. Insects.

[31] Liu Y., Zhao Q., Wang X., Sheng Y., Tian W., Ren Y. (2024). A tree species classification model based on improved YOLOv7 for shelterbelts. Front. Plant Sci..

[32] Wang S., Li Y., Qiao S. (2024). ALF-YOLO: Enhanced YOLOv8 based on multiscale attention feature fusion for ship detection. Ocean Eng..

[33] Fang C., Li C., Yang P., Kong S., Han Y., Huang X., Niu J. (2024). Enhancing Livestock Detection: An Efficient Model Based on YOLOv8. Appl. Sci..

[34] Elshawi R., Maher M., Sakr S. (2019). Automated machine learning: State-of-the-art and open challenges. arXiv.

[35] Feurer M., Klein A., Eggensperger K., Springenberg J., Blum M., Hutter F. Efficient and robust automated machine learning. Proceedings of the Advances in Neural Information Processing Systems Annual Conference.

[36] Bischl B., Binder M., Lang M., Pielok T., Richter J., Coors S., Thomas J., Ullmann T., Becker M., Boulesteix A.L. (2023). Hyperparameter optimization: Foundations, algorithms, best practices, and open challenges. Wiley Interdiscip. Rev. Data Min. Knowl. Discov..

[37] Bergstra J., Yamins D., Cox D. Making a science of model search: Hyperparameter optimization in hundreds of dimensions for vision architectures. Proceedings of the 30th International Conference on Machine Learning (PMLR).

[38] Alibrahim H., Ludwig S.A. Hyperparameter optimization: Comparing genetic algorithm against grid search and bayesian optimization. Proceedings of the 2021 IEEE Congress on Evolutionary Computation (CEC).

[39] Yang L., Shami A. (2020). On hyperparameter optimization of machine learning algorithms: Theory and practice. Neurocomputing.

[40] Bergstra J., Bengio Y. (2012). Random search for hyper-parameter optimization. J. Mach. Learn. Res..

[41] Ramos L., Casas E., Bendek E., Romero C., Rivas-Echeverría F. (2024). Hyperparameter optimization of YOLOv8 for smoke and wildfire detection: Implications for agricultural and environmental safety. Artif. Intell. Agric..

[42] Solimani F., Cardellicchio A., Dimauro G., Petrozza A., Summerer S., Cellini F., Renò V. (2024). Optimizing tomato plant phenotyping detection: Boosting YOLOv8 architecture to tackle data complexity. Comput. Electron. Agric..

[43] Lee Y.-S., Patil M.P., Kim J.G., Choi S.S., Seo Y.B., Kim G.-D. (2024). Improved Tomato Leaf Disease Recognition Based on the YOLOv5m with Various Soft Attention Module Combinations. Agriculture.

[44] Khan Q. (2022). Tomato Disease Multiple Sources.

[45] Khanam R., Hussain M. (2024). YOLOv11: An overview of the key architectural enhancements. arXiv.

[46] Sapkota R., Meng Z., Churuvija M., Du X., Ma Z., Karkee M. (2024). Comprehensive performance evaluation of yolo11, yolov10, yolov9 and yolov8 on detecting and counting fruitlet in complex orchard environments. arXiv.

[47] Ultralytics (2023). Ultralytics YOLO Docs. https://docs.ultralytics.com/.

[48] Redmon J., Farhadi A. (2018). YOLOv3: An incremental improvement. arXiv.

[49] Kandel I., Castelli M. (2020). The effect of batch size on the generalizability of the convolutional neural networks on a histopathology dataset. ICT Express.

[50] Czitrom V. (1999). One-factor-at-a-time versus designed experiments. Am. Stat..

[51] Schmeiser B. (1982). Batch size effects in the analysis of simulation output. Oper. Res..

[52] Radiuk P.M. (2017). Impact of training set batch size on the performance of convolutional neural networks for diverse datasets. Inform. Technol. Manag. Sci..

[53] He F., Liu T., Tao D. Control batch size and learning rate to generalize well: Theoretical and empirical evidence. Proceedings of the Advances in Neural Information Processing Systems Annual Conference (NeurIPS 2019).

[54] Takase T., Oyama S., Kurihara M. (2018). Effective neural network training with adaptive learning rate based on training loss. Neural Netw..

[55] Keskar N.S., Mudigere D., Nocedal J., Smelyanskiy M., Tang P.T.P. (2016). On large-batch training for deep learning: Generalization gap and sharp minima. arXiv.

[56] Loshchilov I., Hutter F. (2017). Fixing weight decay regularization in adam. arXiv.

[57] Krogh A., Hertz J. A simple weight decay can improve generalization. Proceedings of the Advances in Neural Information Processing Systems Annual Conference.

[58] Qian N. (1999). On the momentum term in gradient descent learning algorithms. Neural Netw..

[59] Sutskever I., Martens J., Dahl G., Hinton G. On the importance of initialization and momentum in deep learning. Proceedings of the 30th International Conference on Machine Learning (PMLR).

[60] Dozat T. Incorporating Nesterov Momentum into Adam. Proceedings of the 4th International Conference on Learning Respresentations, Workshop Track.

[61] Srivastava N., Hinton G., Krizhevsky A., Sutskever I., Salakhutdinov R. (2014). Dropout: A simple way to prevent neural networks from overfitting. J. Mach. Learn. Res..

[62] Ko B., Kim H.-G., Oh K.-J., Choi H.-J. Controlled dropout: A different approach to using dropout on deep neural network. Proceedings of the 2017 IEEE International Conference on Big Data and Smart Computing (BigComp).

[63] Baldi P., Sadowski P.J. Understanding dropout. Proceedings of the Advances in Neural Information Processing Systems Annual Conference.

[64] Brownlee J. (2018). What is the Difference Between a Batch and an Epoch in a Neural Network. Mach. Learn. Mastery.

[65] Hastomo W., Karno A.S.B., Kalbuana N., Meiriki A. (2021). Characteristic parameters of epoch deep learning to predict COVID-19 data in Indonesia. J. Phys. Conf. Ser..

[66] Komatsuzaki A. (2019). One epoch is all you need. arXiv.

[67] Hoffer E., Hubara I., Soudry D. (2018). Train longer, generalize better: Closing the generalization gap in large batch training of neural networks. arXiv.

[68] Meng C., Liu S., Yang Y. DMAC-YOLO: A High-Precision YOLO v5s Object Detection Model with a Novel Optimizer. Proceedings of the 2024 International Joint Conference on Neural Networks (IJCNN).

[69] Li S., Sun S., Liu Y., Qi W., Jiang N., Cui C., Zheng P. (2024). Real-time lightweight YOLO model for grouting defect detection in external post-tensioned ducts via infrared thermography. Autom. Constr..

[70] Arora S., Dalal S., Sethi M.N. Interpretable features of YOLO v8 for Weapon Detection-Performance driven approach. Proceedings of the 2024 International Conference on Emerging Innovations and Advanced Computing (INNOCOMP).

[71] Santos C., Aguiar M., Welfer D., Belloni B. (2022). A new approach for detecting fundus lesions using image processing and deep neural network architecture based on YOLO model. Sensors.

[72] Goodfellow I. (2016). Deep Learning.

[73] Li H., Xu Z., Taylor G., Studer C., Goldstein T. Visualizing the loss landscape of neural nets. Proceedings of the Advances in Neural Information Processing Systems Annual Conference.

[74] Wan R., Zhu Z., Zhang X., Sun J. Spherical motion dynamics: Learning dynamics of normalized neural network using sgd and weight decay. Proceedings of the Advances in Neural Information Processing Systems Annual Conference.

[75] Nakamura K., Hong B.-W. (2019). Adaptive weight decay for deep neural networks. IEEE Access.

[76] Cutkosky A., Mehta H. Momentum improves normalized sgd. Proceedings of the 37th International Conference on Machine Learning, PMLR.

[77] Yu H., Jin R., Yang S. On the linear speedup analysis of communication efficient momentum SGD for distributed non-convex optimization. Proceedings of the 36th International Conference on Machine Learning, PMLR.

[78] Smith L.N. (2017). Cyclical learning rates for training neural networks. arXiv.

